# Interleukin-17A-promoted MSC2 polarization related with new bone formation of ankylosing spondylitis

**DOI:** 10.18632/oncotarget.20823

**Published:** 2017-09-11

**Authors:** Tao He, Yan Huang, Chen Zhang, Denghui Liu, Chao Cheng, Weidong Xu, Xiaoling Zhang

**Affiliations:** ^1^ Department of Joint Surgery and Sports Medicine, Changhai Hospital Affiliated to the Second Military Medical University, Shanghai, People’s Republic of China; ^2^ The Key Laboratory of Stem Cell Biology, Institute of Health Sciences, Shanghai Jiao Tong University School of Medicine, Shanghai Institutes for Biological Sciences, Chinese Academy of Sciences, Shanghai, People’s Republic of China; ^3^ Department of Orthopedic Surgery, Xin Hua Hospital Affiliated to Shanghai Jiao Tong University School of Medicine, Shanghai, People’s Republic of China; ^4^ Department of Nuclear Medicine, Changhai Hospital Affiliated to the Second Military Medical University, Shanghai, People’s Republic of China

**Keywords:** interleukin-17, mesenchymal stem cells, polarization, new bone formation, ankylosing spondylitis

## Abstract

It’s still unknown how over-hyperplasia of tissue such like new bone formation (NBF) developed in ankylosing spondylitis (AS). We found low level of IL-17A promoted TLR4+MSC1 polarization with suppressed osteogenic differentiation through JAK2/STAT3 pathway, while high level of IL-17A promoted TLR3+MSC2 polarization with enhanced osteogenic differentiation through WNT10b/RUNX2 pathway. Furthermore, both proteoglycan-induced spondylitis (PGISp) mouse model and AS patients without NBF showed MSC1 polarization, up-regulated JAK2/STAT3 pathway and high level of IL-17A (peripherally, but not locally), but those with NBF showed MSC2 polarization, up-regulated WNT10b/RUNX2 pathway and high expression of IL-17A at local site. Results showed NBF of AS was induced by MSC2 polarization that was promoted by high level of IL-17A, and may be treated by suppressing local MSC2 polarization.

## INTRODUCTION

Many chronic inflammatory diseases have extensive tissue hyperplasia which makes pathological changes more complicated. Ankylosing spondylitis (AS), the major subtype of spondyloarthritis (SpA), is such an immune-mediated disease and characterized by spinal inflammation and ankylosis, peripheral arthritis, enthesopathy, extra-articular manifestations (i.e. uveitis, psoriasis, inflammatory bowel disease and cardiovascular disease) and a strong genetic association [1, 2]. The main clinical features of SpA are inflammatory tissue destruction, but the most characteristic features of AS was osteoproliferation and ankylosis that lead to irreversible ossification, the loss of mobility and deformity in late phase. Therefore, the two central features of AS are inflammation and new bone formation (NBF). However, it’s still unknown how NBF developed in AS [1].

Mesenchymal stem cells (MSCs) had both proinflammatory and regenerative effect, that is to say, MSCs may have capablity of polarized differentiation [3, 4]. MSCs that expressed surface marker Toll-like receptor (TLR) 4 can be activated by products of bacteria infection or tissue damage (i.e. lipopolysaccharides or proteoglycan) [3, 5]. TLR4+MSCs can secrete proinflammatory cytokine and chemokines C-C motif ligand (CCL) 5 [6, 7] to promote proinflammatory macrophage, which can secrete proinflammatory IL-23 to activate T helper 17 cells (Th17) through JAK2/STAT3 pathway [8, 9]. Therefore, TLR4+MSCs (or called MSC1) may promote early or mild phase of peripheral inflammation directly or indirectly (through enhancing immunal response). However, in late or severe phase of inflammation, TLR3+MSCs (or called MSC2) become activated by double-stranded ribonucleic acid (dsRNA) (products of cell damage) [4]. MSC2 may promote anti-inflammatory macrophage (producing interleukin-10) [10] and T regulatory cells (Tregs) by secreting high level of transforming growth factor beta (TGF-β) [11], chemokines C-X-C motif (CXCL) 10 and prostaglandin E2 (PGE_2_) [4]. Tregs can suppress Th17 [3, 7]. Therefore, high level of proinflammatory cytokine may promote anti-inflammatory MSC2, and induce decreased peripheral inflammation and local over-hyperplasia of tissue such like NBF of AS, but it hasn’t been proved.

Interleukin-17A (IL-17A) is one of the key inflammatory cytokines primarily produced by Th17 in infectious, inflammatory, and autoimmune responses like AS by targeting cells to produce proinflammatory cytokines such as tumor necrosis factor (TNF). It was pathological origin of ossification of psoriatic arthritis [8]. Previous study showed cell damage induced by IL-17A produced extracellular endogenous dsRNA in autoimmune responses [12]. High level (20 ng/ml) of recombinant mouse IL-17A dramatically enhanced the immunosuppressive effect of murine MSCs [13], and higher level (50 ng/ml) of recombinant human IL-17A increased osteogenic differentiation of human MSCs by inhibiting Dickkopf 1 (DKK-1) (a WNT pathway inhibitor) mRNA expression [14]. Therefore, high level of cytokine IL-17A may be produced by MSC1 polarization in early inflammatory phase of AS, and it may activate osteogenic differentiation of TLR3+MSC2 through WNT pathway and induce NBF in late phase of AS. Based on results of previous studies, we hypothesized that high level of IL-17A promoted MSC2 polarization that induced NBF of AS.

## RESULTS

### High level of IL-17A promoted MSC2 polarization *in vitro*

**Figure 1:**
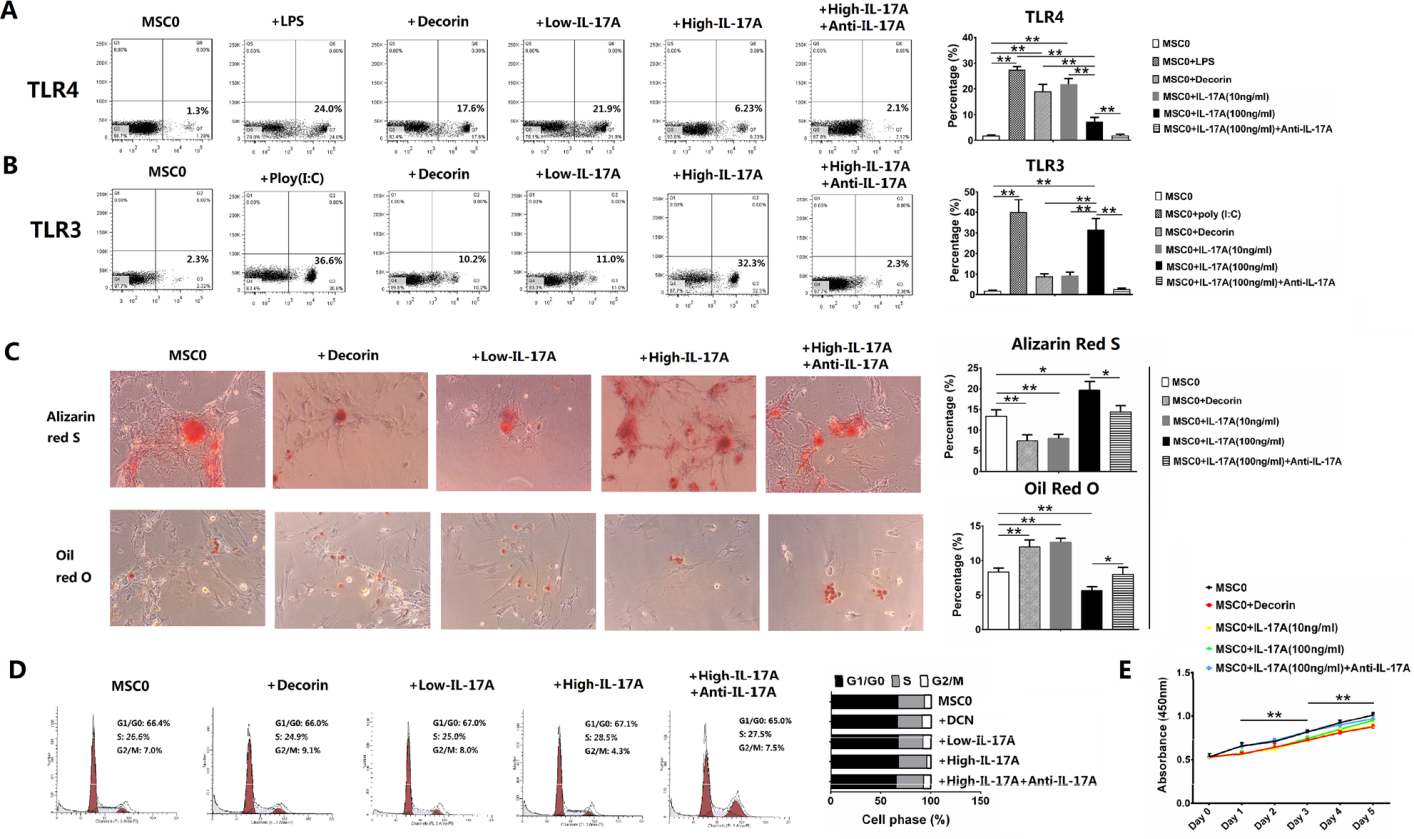
IL-17A mediated MSC1 and MSC2 polarizations in concentration-dependent way Values are the mean ± SEM (all *n* = 3). ^*^P < 0.05, ^**^P < 0.01. (**A**–**B**) Flow cytometry of murine MSCs. (**C**) Osteogenic and adipocytic differentiation of murine MSCs. (**D**) Distribution percentages of murine MSCs in G0/G1, S and G2/M phases of the cell cycle. (**E**) Cellular proliferation assay of murine MSCs .

To investigate modulatory role of IL-17A on MSC polarizations, we used murine bone marrow-derived MSCs (mBMSCs) which was CD44+ (86.1%), CD105+ (97.7%), CD90.2+ (58.4%), CD34– (0.5%), CD40L–(2.1%), CD45– (1.6%) without stimulation (MSC0) in flow cytometry (Supplementary Figure 1A). Our results showed the expression of TLR4 of MSCs was significantly higher after stimulation of lipopolysaccharides (LPS), decorin or 10 ng/ml IL-17A (Low-IL-17A) than both in MSC0 and after stimulation of 100 ng/ml IL-17A (High-IL-17A) (all *p <* 0.01, Figure 1A). However, the expression of TLR3 was significantly higher after stimulation of polyinosinic: polycytidylic acid [Poly(I:C)], a synthetic dsRNA viral mimic, or 100 ng/ml IL-17A than in MSC0 or after stimulation of decorin or 10 ng/ml IL-17A (all *p <* 0.01, Figure 1B). The expression of both TLR4 and TLR3 was significantly higher after stimulation of 100 ng/ml IL-17A than after adding anti-IL-17A to 100 ng/ml IL-17A (both *p <* 0.01, Figure 1A and 1B). We found Allison blue staining in chondrocytic differentiation had no differences between before and after stimulation of decorin, 10 or 100 ng/ml IL-17A (Supplementary Figure 1C). However, Alizarin red S staining in osteogenic differentiation were significantly weaker and Oil Red O staining in adipocytic differentiation was significantly stronger in MSCs after stimulation of decorin or 10 ng/ml IL-17A than in MSC0 (all *p <* 0.01, Figure 1C). And Alizarin red S staining was significantly stronger and Oil Red O staining was significantly weaker in MSCs after stimulation of 100 ng/ml IL-17A than both in MSC0 (*p <* 0.05 and 0.01, respectively) and in MSCs after adding anti-IL-17A (both *p <* 0.05) (Figure 1C). MSC1 and MSC2 (after stimulation of 10 and 100 ng/ml IL-17A, respectively) had no differences in appearances (Supplementary Figure 1B), and most of MSCs before and after stimulation of decorin, 10 or 100 ng/ml IL-17A and inhibition of anti-IL-17A were in G0/G1 phase of the cell cycle in flow cytometry (Figure 1D). Cellular proliferating capability of MSCs was significantly better on day 3 than on day 1, and on day 5 than on day 3 before and after stimulation of decorin, 10 or 100 ng/ml IL-17A, or adding anti-IL-17A (all *p <* 0.01, Figure 1E). All these results showed low and high level of IL-17A promoted MSC1 and MSC2 polarizations which had suppressed and promoted osteogenic differentiations, respectively.

### High level of IL-17A promoted MSC2 polarization through WNT10b/RUNX2 pathway

**Figure 2:**
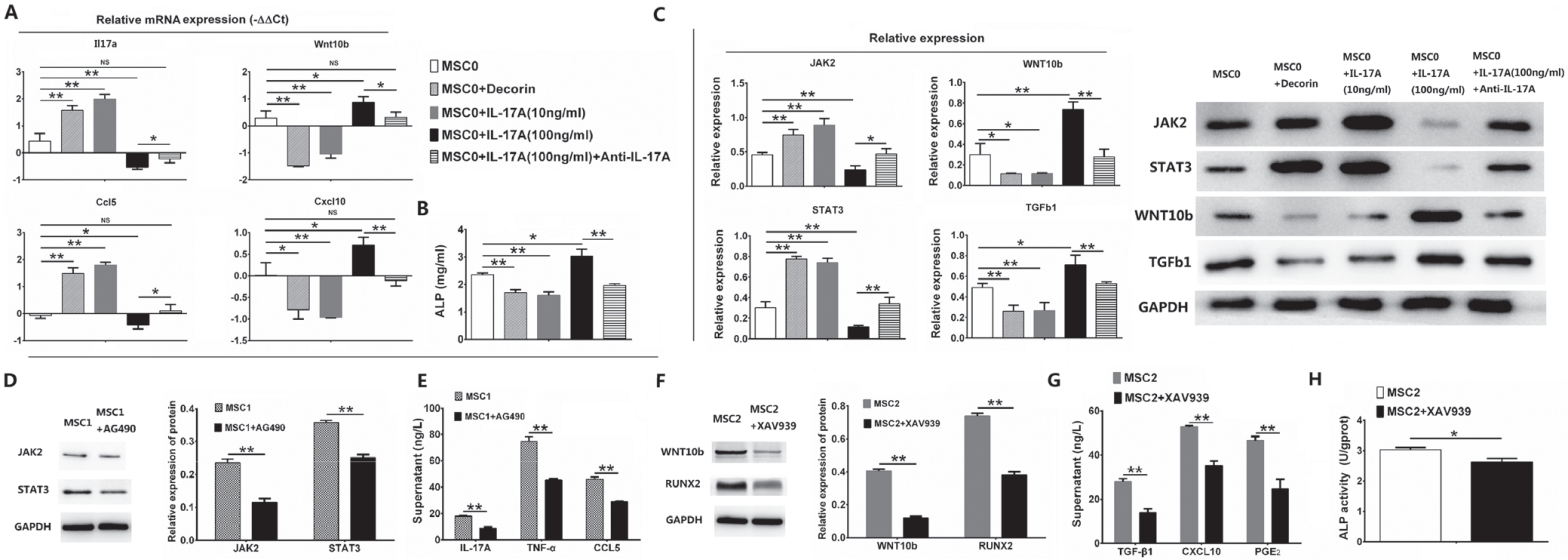
Signal pathways of MSC1 and MSC2 polarizations Values are the mean ± SEM (all *n* = 3). ^*^P < 0.05, ^**^P < 0.01. (**A**) The relative mRNA expression of murine MSCs in RT-qPCR for Il17a, Ccl5, Cxcl10 and Wnt10b. (**B**) ALP acitivity for murine MSCs. (**C**) The relative protein expression of murine MSCs in western blotting for JAK2, STAT3, WNT10b and TGFb1. (**D**) The relative protein expression of MSC1 in western blotting for JAK2 and STAT3 after adding AG490 (inhibitor of JAK2). (**E**) The supernatant analysis of MSC1 in Elisa for IL-17A, TNF-α and CCL5 after adding AG490. (**F**) The relative protein expression of MSC2 in western blotting for WNT10b and RUNX2 after adding XAV939 (inhibitor of WNT10b). (**G**) The supernatant analysis of MSC1 in Elisa for TGF-β1, CXCL10 and PGE_2_ after adding XAV939. (**H**) ALP acitivity of MSC2 after adding XAV939.

It’s reported that receptors of IL-17A and TLR4 had common combining site and activated the same down-stream inflammatory signal pathway [15], and we found low level of IL-17A promoted TLR4+MSC (Figure 1A). Furthermore, TLR4 possibly activate down-stream JAK/STAT pathway to secrete IL-17A in inflammatory bowel disease [16], which suppressed WNT pathway through stimulating DKK1 in intestinal stem cells [17, 18]. However, TLR3 was able to activate down-stream WNT pathway of hair follicle stem cells [19] that also was important pathway of osteogenic differentiaion of MSCs [14, 20] and can suppress inflammation through suppressing STAT3 in the uterine mesenchyme [21] and promoting TGFb/Smad pathway in MSCs [22]. Therefore, we want to find if MSC1 and MSC2 polarizations were promoted by low and high level of IL-17A through JAK/STAT and WNT pathway, respectively. Our results of the relative mRNA expression showed both Il17a and Ccl5 were significantly higher, and both Wnt10b and Cxcl10 were significantly lower in MSCs after stimulation of decorin (*p <* 0.01, 0.01, 0.01 and 0.05, respectively) or 10 ng/ml IL-17A (all *p <* 0.01) than in MSC0 (Figure 2A). However, both Il17a and Ccl5 were significantly lower, and both Wnt10b and Cxcl10 were significantly higher in MSCs after stimulation of 100 ng/ml IL-17A than in MSC0 (*p <* 0.01, 0.05, 0.05 and 0.05, respectively, Figure 2A). And both Il17a and Ccl5 were significantly higher, and both Wnt10b and Cxcl10 were significantly lower in MSCs after stimulation of 100 ng/ml IL-17A than in MSCs after adding anti-IL-17A (*p <* 0.05, 0.05, 0.05 and 0.01, respectively, Figure 2A). Furthermore, the activity of alkaline phosphatase (ALP) was significantly lower in MSCs after stimulation of decorin or 10 ng/ml IL-17A (both *p <* 0.01) than in MSC0, and significantly higher in MSCs after stimulation of 100 ng/ml IL-17A than both in MSC0 and in MSCs after adding anti-IL-17A (*p <* 0.05 and 0.01, respectively) (Figure 2B). Analysis of western blotting (WB) showed both JAK2 and STAT3 had significantly higher, and both WNT10b and TGFb1 had significantly lower expression in MSCs after stimulation of decorin or 10 ng/ml IL-17A (*p <* 0.01, 0.01, 0.05 and 0.01, respectively) than in MSC0 (Figure 2C). However, both JAK2 and STAT3 had significantly lower, and both WNT10b and TGFb1 had significantly higher expression in MSCs after stimulation of 100 ng/ml IL-17A than both in MSC0 (*p <* 0.01, 0.01, 0.01 and 0.05, respectively) and in MSCs after adding anti-IL-17A (*p <* 0.05, 0.01, 0.01 and 0.01, respectively) (Figure 2C). Finally, we found both JAK2 and STAT3 had significantly lower expression in MSCs after adding AG490 (inhibitor of JAK2) to MSC1 than in MSC1 alone (both *p <* 0.01, Figure 2D), but both WNT10b and RUNX2 had significantly lower expression in MSCs after adding XAV939 (inhibitor of WNT10b) to MSC2 than in MSC2 alone in WB (both *p <* 0.01, Figure 2F). And the level of supernatant IL-17A, TNF-α and CCL5 of MSC1 in Elisa were significantly lower in MSCs after adding AG490 than in MSC1 alone (all *p <* 0.01, Figure 2E). The level of supernatant TGF-β1, CXCL10 and PGE_2_, and ALP activity of MSC2 in Elisa were significantly lower in MSCs after adding XAV939 than in MSC2 alone (*p <* 0.01, 0.01, 0.01 and 0.05, respectively, Figure 2G and 2H). All these results showed low level of IL-17A promoted MSC1 polarization and secretion of IL-17A, TNF-α and CCL5 through JAK2/STAT3 pathway, while high level of IL-17A promoted MSC2 polarization, osteogenic effects and secretion of TGF-β1, CXCL10 and PGE_2_ through WNT10b/RUNX2 pathway.

### MSC2 polarization promoted M2 and Treg polarizations in coculture analysis

**Figure 3:**
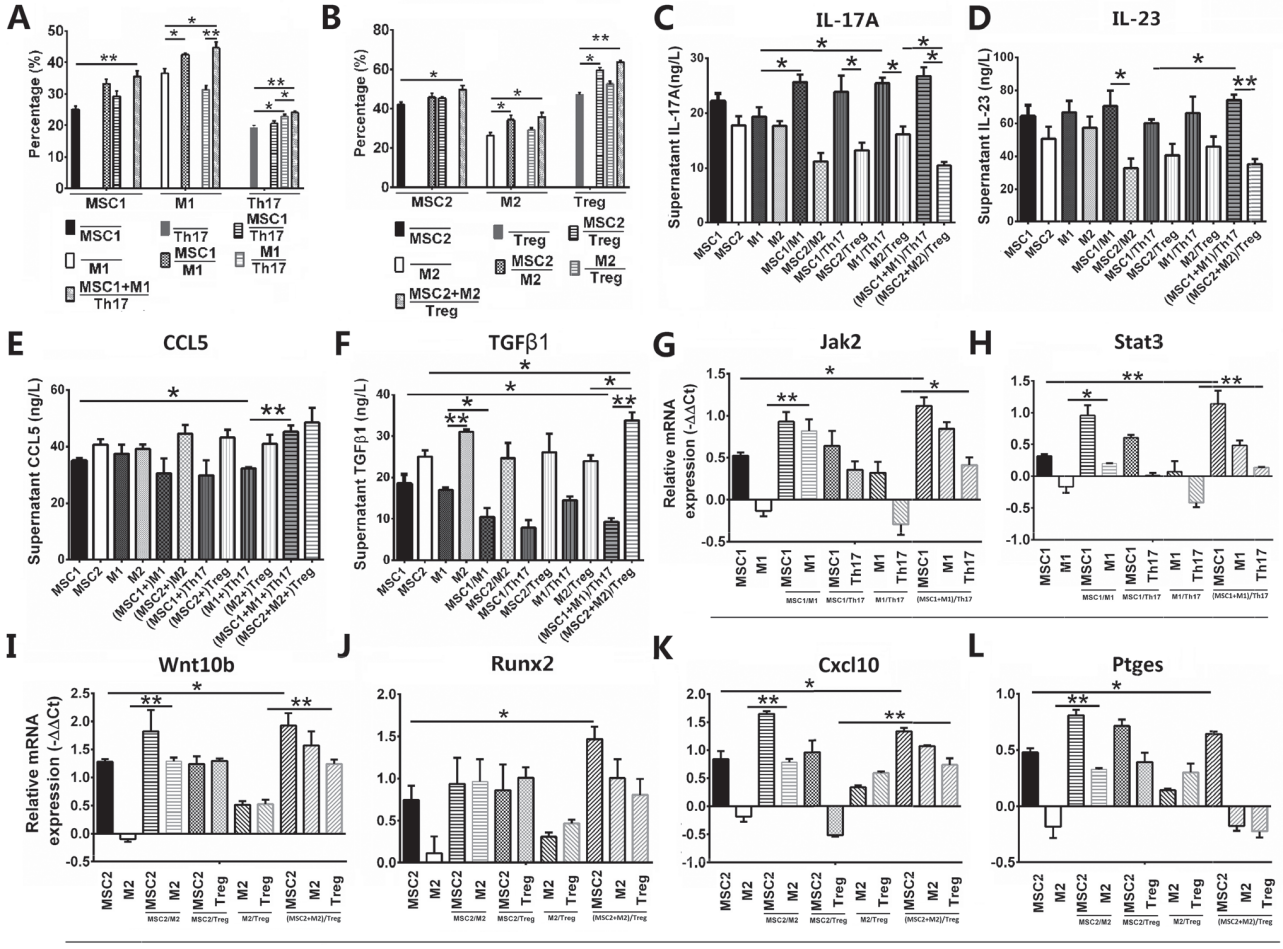
MSC1/2 polarizations promoted M1/2 and Th17/Treg polarizations in coculture analysis, respectively Values are the mean ± SEM (all *n* = 3). ^*^P < 0.05, ^**^P < 0.01. (**A**) Flow cytometry of MSC1, M1 and Th17 as monocultures or co-culture. (**B**) Flow cytometry of MSC2, M2 and Treg as monocultures or co-culture. (**C**–**F**) The analysis for supernant IL-17A (C), IL-23 (D), CCL5 (E) and TGF-β1 (F) in Elisa. (**G**–**L**) The relative mRNA expression of Jak2 (G), Stat3 (H), Wnt10b (I), Runx2 (J), Cxcl10 (K) and Ptges (L) in RT-qPCR.

It’s reported that IL-17A is primarily produced by Th17 that is mainly activated by IL-23 produced by proinflammatory macrophage [8], macrophage had proinflammatory type 1 macrophage (M1) and anti-inflammatory type 2 macrophage (M2) polarizations [4, 10], and T cells (Tc) had Th17 and Treg polarizations [11]. We want to know if MSC1 polarization enhanced high level of IL-17A through promoting M1 or Th17 polarizations, and MSC2 polarization suppressed IL-17A through promoting M2 or Treg polarizations. Our results showed after stimulation of LPS and IL-4 murine M1 and M2 had no differences in appearances (Supplementary Figure 2A), but had significantly higher expression of F4/80 (44.73%) and both F4/80 and CD206 (38.55%) than M0 (without stimulation) (2.92% and 2.71%) in flow cytometry (both *p <* 0.01, Supplementary Figure 2B and 2C), respectively. M1 had significantly higher absorbance in cellular phagocytosis assay and stronger tartrate-resistant acid phosphatase (TRAP) staining than both M0 and M2 (all *p <* 0.01, orignal magnification 100×, Supplementary Figure 2D and 2E). Murine CD4+T cells isolated from spleen (without stimulation) expressed very low IL-17A and CD25 (both < 2%) (data not showed). After stimulation of 30 ng/ml IL-23 with 2 ng/ml TGF-β1, and 5 ng/ml TGF-β1, Tc was both CD4+ (95.0%) and IL17+ (60.2%), and both CD4+ (87.8%) and CD25+ (57.1%), respectively (Supplementary Figure 2F). Furthermore, MSC1/2 and M1/2 were cultured in transwell lower chamber as monocultures, or co-cultured in upper chamber as monocultures or co-cultures with Th17/Treg in low chamber for two days, respectively. Our results showed MSC1 significantly increased after coculturing with both M1 and Th17 in incontact way (*p <* 0.01), M1 significantly increased after coculturing with MSC1 in both contact and incontact way (both *p <* 0.05), and Th17 significantly increased after coculturing with M1 in incontact way (*p <* 0.05) in flow cytometry (Figure 3A). However, MSC2 significantly increased after coculturing with both M2 and Treg in incontact way (*p <* 0.05), M2 significantly increased after coculturing with MSC2 in both contact and incontact way (both *p <* 0.05), and Treg significantly increased after coculturing with MSC2 in incontact way (*p <* 0.05) (Figure 3B). These results showed MSC1 promoted Th17 through promoting M1, and was promoted by both Th17 and M1 in incontact way, while MSC2 promoted both M2 and Treg, and was promoted by both M2 and Treg in incontact way.

Since MSC1/2 polarizations interacted M1/2 and Th17/Treg polarizations in incontact way, we next investigated the secretory factors in supernatants. Our results showed the supernatant IL-17A was significantly higher in M1 than in M1 cocultured with MSC1 or Th17, in M2 cocultured with Treg alone than in M2 cocultured with both Treg and MSC2, and in Th17 cocultured with MSC1, M1 or both than in Treg cocultured with MSC2, M2 or both, respectively (all *p <* 0.05, Figure 3C). The supernatant IL-23 was significantly higher in MSC1 cocultured with both M1 and Th17 than in MSC1 cocultured with Th17 alone (*p <* 0.05), in M1 cocultured with MSC1 than in M2 cocultured with MSC2 (*p <* 0.05), and in Th17 cocultured with both M1 and MSC1 than in Treg cocultured with both M2 and MSC2 (*p <* 0.01) (Figure 3D). The supernatant TNF-α was significantly higher in MSC1 cocultured with M1 than in M1 alone (*p <* 0.01), in M1 cocultured with Th17 than in M1 alone (*p <* 0.05), and in Treg cocultured with M2 than in MSC2 cocultured with both Treg and M2, and in MSC1 or M1 alone than in MSC2 or M2 alone, respectively (all *p <* 0.01) (Supplementary Figure 3A). The supernatant CCL5 was significantly higher in MSC1 alone than in M1 cocultured with Th17 (*p <* 0.05), and in M1 cocultured with both Th17 and MSC1 than in M1 cocultured with Th17 alone (*p <* 0.01) (Figure 3E). However, supernatant TGF-β1 was significantly lower in M1 alone than in M2 alone (*p <* 0.01), in MSC1 cocultured with both M1 and Th17 than in MSC1 alone (*p <* 0.05), in MSC1 cocultured with M1 than in MSC1 alone (*p <* 0.05), in Treg cocultured with M2 than in Treg cocultured with both M2 and MSC2 (*p <* 0.05), and in Th17 cocultured with both MSC1 and M1 than in Treg cocultured with both MSC2 and M2 (*p <* 0.01) (Figure 3F). All these results showed MSC1 primed by low level of IL-17A secreted IL-17A and promoted IL-17A-producing Th17 by promoting IL-23-producing M1. On the contrary, MSC2 primed by high level of IL-17A secreted TGF-β1, and promoted TGFβ1-producing M2 and Treg.

Furthermore, we made analysis for transductional signals and found the relative mRNA expression of Jak2 and Stat3 was significantly higher in MSC1 cocultured with both M1 and Th17 than in MSC1 alone (*p <* 0.05 and 0.01, respectively), in M1 cocultured with MSC1 than in M1 alone (*p <* 0.01 and 0.05, respectively), and in Th17 cocultured with both MSC1 and M1 than in Th17 cocultured with M1 alone (*p <* 0.05 and 0.01, respectively) (Figure 3G and 3H). The relative mRNA expression of Nfkb1 was only significantly higher in M1 cocultured with MSC1 than in M1 alone (*p <* 0.01), and in Th17 cocultured with both MSC1 and M1 than in Th17 cocultured with M1 alone (*p <* 0.05) (Supplementary Figure 3B). However, the relative mRNA expression of Wnt10b was significantly higher in MSC2 cocultured with both M2 and Treg than in MSC2 alone (*p <* 0.05), in M2 cocultured with MSC2 than in M2 alone (*p <* 0.01), and in Treg cocultured with both MSC2 and M2 than in Treg with M2 alone (*p <* 0.01) (Figure 3I). The relative mRNA expression of Runx2 was significantly higher in MSC2 cocultured with both M2 and Treg than in MSC2 alone (*p <* 0.05, Figure 3J). Wnt5a had no significantly higher mRNA expression in MSC2, M2 or Treg (Supplementary Figure 3C). The relative mRNA expression of Cxcl10 was significantly higher in MSC2 cocultured with both M2 and Treg than in MSC2 alone (*p <* 0.05), in M2 cocultured with MSC2 than in M2 alone (*p <* 0.01), and in Treg cocultured with both MSC2 and M2 than in Treg cocultured with MSC2 alone (*p <* 0.01) (Figure 3K). The relative mRNA expression of Ptges was significantly higher in MSC2 cocultured with both M2 and Treg than in MSC2 alone (*p <* 0.05), and in M2 cocultured with MSC2 than in M2 alone (*p <* 0.01) (Figure 3L). It’s reported indoleamine 2,3-dioxygenase (IDO) was another immune-suppressive factor produced by MSC2 [4]. However, the relative mRNA expression of Ido1 was only significantly higher in M2 cocultured with MSC2 than in M2 alone (*p <* 0.01, Supplementary Figure 3D). All these results showed MSC1 secreted IL-17A and CCL5 to promote M1 through JAK2/STAT3 pathway, and MSC2 secreted TGF-β1 and expressed Cxcl10 and Ptges to promote M2 and Treg through WNT10b/RUNX2 pathway.

### IL-17A-promoted MSC2 polarization related with NBF of PGISp mice

**Figure 4:**
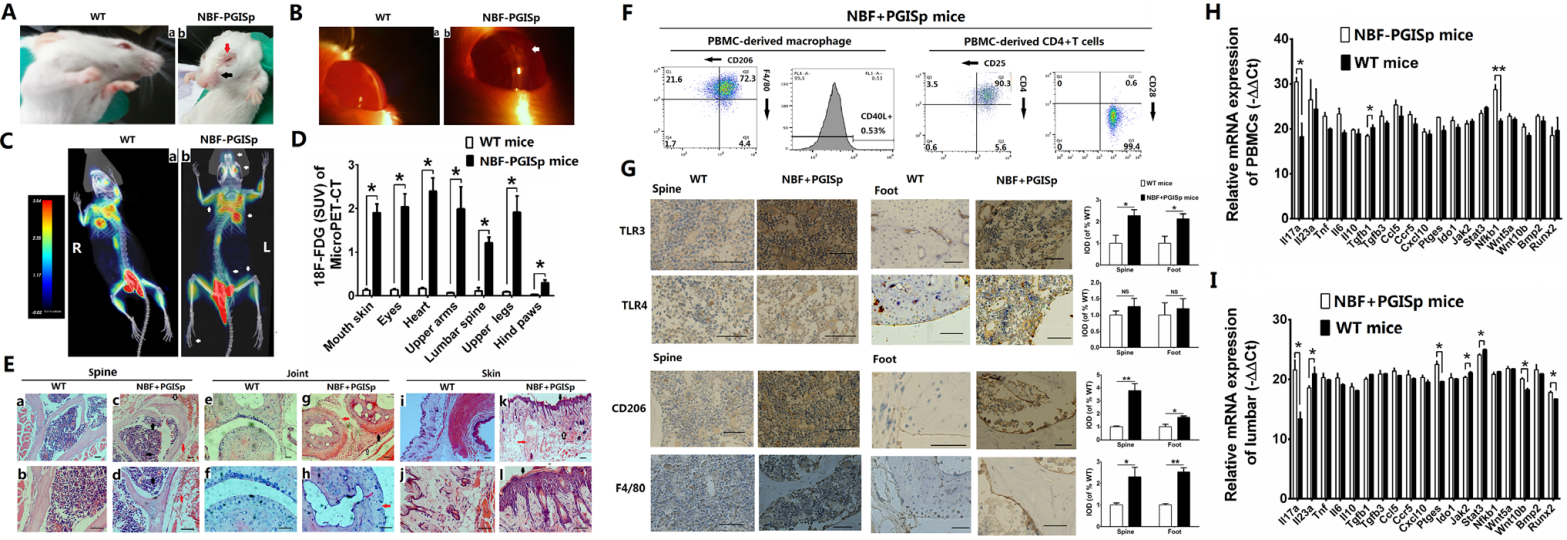
IL-17A-promoted MSC2 polarization related with new bone formation of PGISp mice Values are the mean ± SEM (all *n* = 3). ^*^P < 0.05, ^**^P < 0.01. (**A**) Appearances of PGISp mice without new bone formation (NBF-PGISp) at 5 month old. (**B**) Analysis for eyes of NBF-PGISp mice in slit lamp microscope (Original magnification 400×). (**C**–**D**) MicroPET-CT analysis for NBF-PGISp mice. (**E**) New bone formation and/or tissue hyperplasia of PGISp mice (NBF+PGISp) at 7 month old by HE staining. (**F**) Flow cytometry of PBMCs-derived macrophage and CD4+T cells of NBF+PGISp mice. (**G**) Immunohistochemistry staining for NBF+PGISp mice. (**H**–**I**) The relative mRNA expression of factors related to MSC1/2 polarizations in PBMCs of NBF-PGISp mice (H) and lumbar of NBF+PGISp mice (I) by RT-qPCR.

It’s reported that proteoglycan (PG) was able to induce AS of murine [23, 24], so we used decorin (a small molecule weight of PG) to challenge murine and find if NBF of AS was related to MSC2 polarization promoted by high level of IL-17A. Our results showed the mice after receiving decorin at 3 months old were feed up to 11 months old, but weight was only significantly different at 5 and 6 months old between PG-induced spondylitis (PGISp) and control wild-type (WT) mice (both *p <* 0.05, Supplementary Figure 4A). Although Stiffness score and psoriasis area severity index (PASI) of PGISp mice were significantly higher than those of WT ones at each month (all *p <* 0.01), they increased significantly from 7 and 6 months old, respectively (both *p <* 0.05) (Supplementary Figure 4B and 4C, and Supplementary Videos 1–3). Therefore, the PGISp mice after 6 months old were supposed to have NBF (Supplementary Figure 4A–4C).Furthermore, our results showed PGISp without NBF (NBF-PGISp) mice at 5 months old showed significant psoriasis-like skin condition with erythema, scaling and lost moustache (black arrow showed in Panel b, Figure 4A and Supplementary Video 1), and acute inflammation of eyes including blepharoconjunctivitis (red arrow showed in Panel b, Figure 4A) and central keratitis in slit lamp microscope (white arrow showed in Panel b, Original magnification 400×, Figure 4B). And the NBF-PGISp mice showed significantly higher uptake of 2-[18F]fluoro-2-deoxy-D-glucose (18F-FDG) in multiple organs including mouth skin, eyes, heart, upper arms, lumbar spine, upper legs and hind paws than WT ones in micro-positron emission tomography-computer tomography (MicroPET-CT) scan (all *p <* 0.05, Figure 4C and 4D). However, our results showed PGISp with NBF (NBF+PGISp) mice at 7 months old had extensive tissue hyperplasia with chronic inflammation in Hematoxylin-eosin (HE) stain. The lumbar spine showed hyperplasia of ligaments with increased multi nuclear giant cells and hyperemia (white, black and red arrows showed, respectively) (Panel c and d, bar *=* 200 μm and 50 μm, respectively) (Figure 4E). The foot joint showed fibrosis of capsule and ossification of cartilage with inflammatory destruction of articular cartilage (white, black and red arrows showed, respectively) (Panel g and h, bar *=* 200 μm and 50 μm, respectively) (Figure 4E). The mouth skin showed hyperplasia of epidermis and muscular layer with follicular edema and hyperemia (white, black and red arrows showed, respectively) (Panel k and l, bar *=* 100 μm and 50 μm, respectively) (Figure 4E). Furthermore, the eyes showed inflammatory damage of cornea (C) and vitreous body (VB) (black and white arrows showed, respectively) (Panel b, bar *=* 100 μm), iridocyclitis of iris ciliary body (IC) including inflammation of ciliary processes and fibroplasia (white and black arrows showed, respectively) (Panel d, bar *=* 50 μm), and inflammatory damage of palpebral conjunctiva (PC) with fibrosis (white and black arrows showed, respectively) (Panel f, bar *=* 50 μm) (Supplementary Figure 4D). The heart showed damaged atrioventricular valve and ventricular dilatation with atrial wall thickening (white, black and yellow arrows showed, respectively) (Panel b and d, bar *=* 200 and 50 μm, respectively) (Supplementary Figure 4E). All these results showed decorin (that promoted MSC1 polarization) induced the inflammation of AS in early phase and also was able to induce NBF of AS in late phase through other pathway.

Using flow cytometry, our results showed PBMCs-derived macrophages were F4/80+ (76.7%), CD206+ (93.9%) and CD40L– (0.53%), and PBMCs-derived CD4+Tc were CD4+ (95.9%), CD28+ (99.4%) and CD25+ (93.8%) in NBF+PGISp mice at 7 months old (Figure 4F). Immunohistochemistry (IHC) staining showed the integrated optical density (IOD) of TLR3, CD206 and F4/80 was significantly higher in NBF+PGISp mice than in WT mice in both lumbar spine (*p <* 0.01, 0.05 and 0.01, respectively) and foot joint (*p <* 0.01, 0.01 and 0.05, respectively), but IOD of TLR4 had no significant difference between NBF+PGISp and WT mice in both spine and foot (all bar *=* 50 μm, Figure 4G). These results showed MSC2 polarization promoted NBF of AS and suppressed inflammation by promoting M2 and Treg polarizations. Furthermore, our results of RT-qPCR analysis for factors related to MSC polarizations showed PBMCs of NBF-PGISp mice at 5 months old had significantly higher relative mRNA expression of both Il17a and Nfkb1, and significantly lower mRNA expression of Tgfb1 than those of WT ones (*p <* 0.05, 0.01 and 0.05, respectively, Figure 4H). However, the lumbar with NBF of PGISp mice at 7 months old had significantly higher relative mRNA expression of Il17a, Ptges, Wnt10b and Runx2, and significantly lower relative mRNA expression of Il23a, Jak2 and Stat3 than those of WT ones (all *p <* 0.05, Figure 4I). All these results showed decorin induced high expression of IL-17A in early inflammatory phase of AS (without NBF) peripherally, while local high level of IL17A was related to NBF of AS by promoting MSC2 polarization through WNT10/RUNX2 pathway.

### IL-17A-promoted MSC2 polarization related with NBF of AS patients

**Figure 5:**
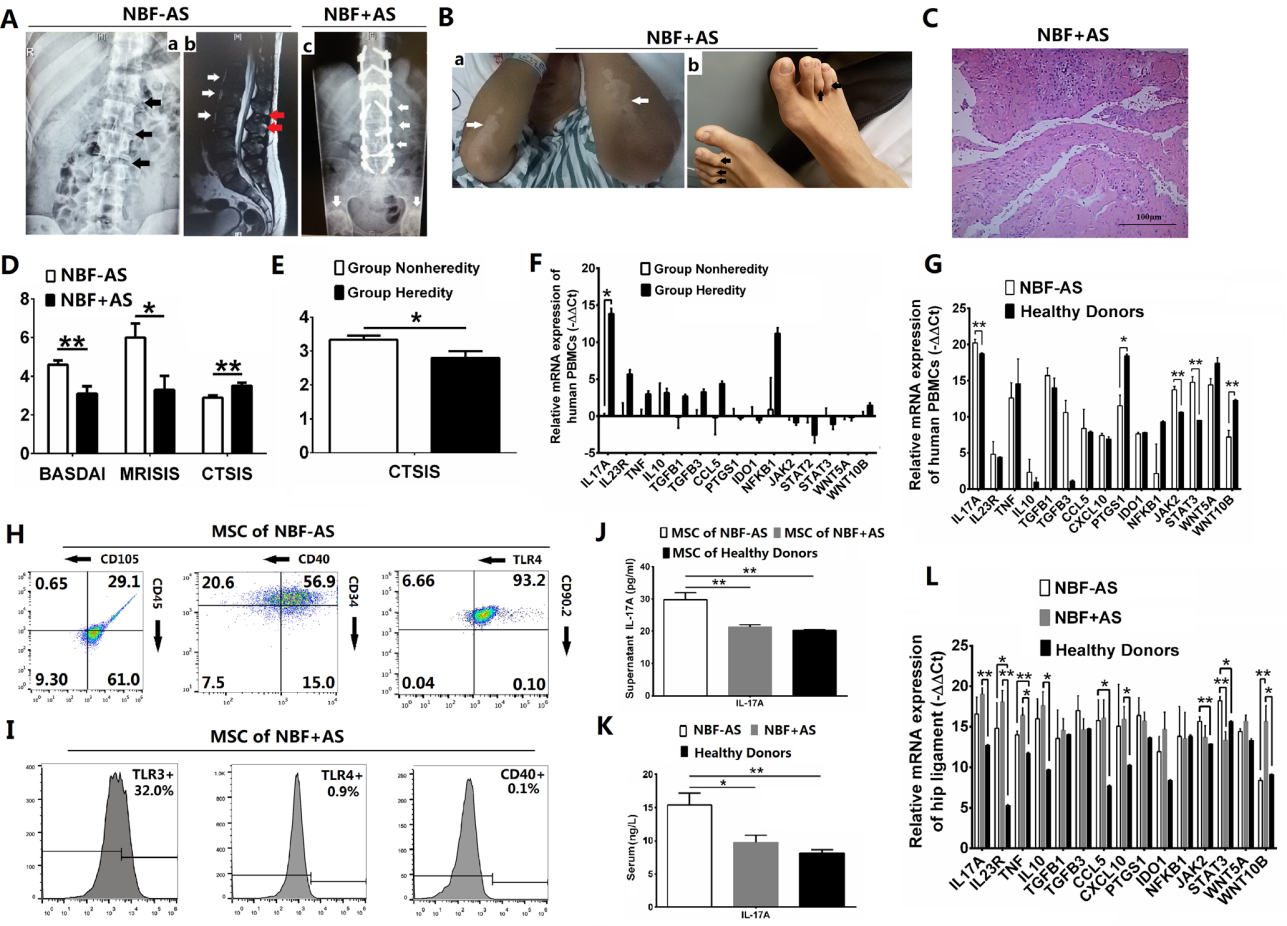
IL-17A-promoted MSC2 polarization related with new bone formation of AS patients Values are the mean ± SEM. ^*^P < 0.05, ^**^P < 0.01. (**A**–**C**) The radiological image of lumbar (A), appearances (B) and HE staining of hip joint (C) of an AS patient. (**D**) BASDAI, MRISIS and CTSIS of NBF+AS patients (both *n* = 10). (**E**) CTSIS of AS patients with and without heredity (*n* = 5 and 15, respectively). (**F**) The relative mRNA expression of factors related to MSC1/2 polarizations in PBMCs of AS patients with and without heredity (both *n*= 3). (**G**) The relative mRNA expression of factors related to MSC1/2 polarizations in PBMCs of NBF-AS patients and healthy donors (both *n* = 3). (**H**–**I**) Flow cytometry of MSCs of NBF– (H) and NBF+ (I) AS patients (both *n* = 5). (**J**–**K**) The analysis for supernant IL-17A of MSCs (J, all *n* = 5) and serum IL-17A (K, all *n* = 10) from AS patients and healthy donors in Elisa. (**L**) The relative mRNA expression of factors related to MSC1/2 polarizations in hip ligament of AS patients and healthy donors (all *n* = 3).

To find if IL-17A-promoted MSC2 polarization related with NBF of AS patients, we made a retrospective analysis at first (Supplementary Table 1). Our results showed the lumbar X ray of an ideal AS patient showed normal lumbar intervertebral space (black arrows showed in Panel a) and his lumbar magnetic resonance imaging (MRI) showed enthesitis of spinal ligament (white and red arrows showed in Panel b, respectively) in 2011 (Figure 5A). In 2014, his X ray showed NBF (bridging symdysmophytes and ankylosis) of lumbar spine with osteotomy and bilateral hip joints (white arrows showed in Panel c, Figure 5A), and he also had psoriasis-like skin lesions and dactylitis (white and black arrows showed in Panel a and b, respectively, Figure 5B). Furthermore, his right hip showed chronic synovitis with degeneration and necrosis of both cartilage and bone in HE stain after total hip arthroplasty (THA) in 2014 (Figure 5C). And pelvic MRI for another AS patient showed inflammatory appearances was mild on the right hip and severe on the left (white arrows showed in Panel a, Supplementary Figure 5A) in 2011. In 2014, his pelvic X-ray showed no obvious damage on the right hip, but significant sclerosis and ankylosis on the left (black arrows showed in Panel b, Supplementary Figure 5A). Furthermore, the group of AS patients without NBF (NBF-AS) had significantly higher Bath ankylosing spondylitis disease activity index (BASDAI) [25] and magnetic resonance imaging sacroiliitis score (MRISIS) [26], and significantly lower computer tomography sacroiliitis score (CTSIS) [27] and complications than the group of AS patients with NBF (NBF+AS) (*p <* 0.01, 0.05, 0.01 and 0.05, respectively, Figure 5D and Supplementary Figure 5B). All these results showed NBF with decreased inflammation in late phase derived from inflammation in early phase of AS.

It’s reported that AS had a strong genetic association [1, 28]. Our results showed AS patients with familial heredity (Group Heredity) had significantly lower CTSIS than those without familial heredity (Group Nonheredity) (*p <* 0.05, Figure 5E). Among factors possibly related to MSC polarized differentiation and AS [1, 3, 4], only IL-17A of PBMCs had significantly higher relative mRNA expression in Group Heredity than in Group Nonheredity (*p <* 0.05, Figure 5F). Furthermore, the relative mRNA expression of IL17A was significantly higher in PBMCs of NBF-AS group than in that of Healthy donors group (*p <* 0.01, Figure 5G). These results showed peripheral inflammation of early AS was related to hereditary IL-17A.

**Figure 6:**
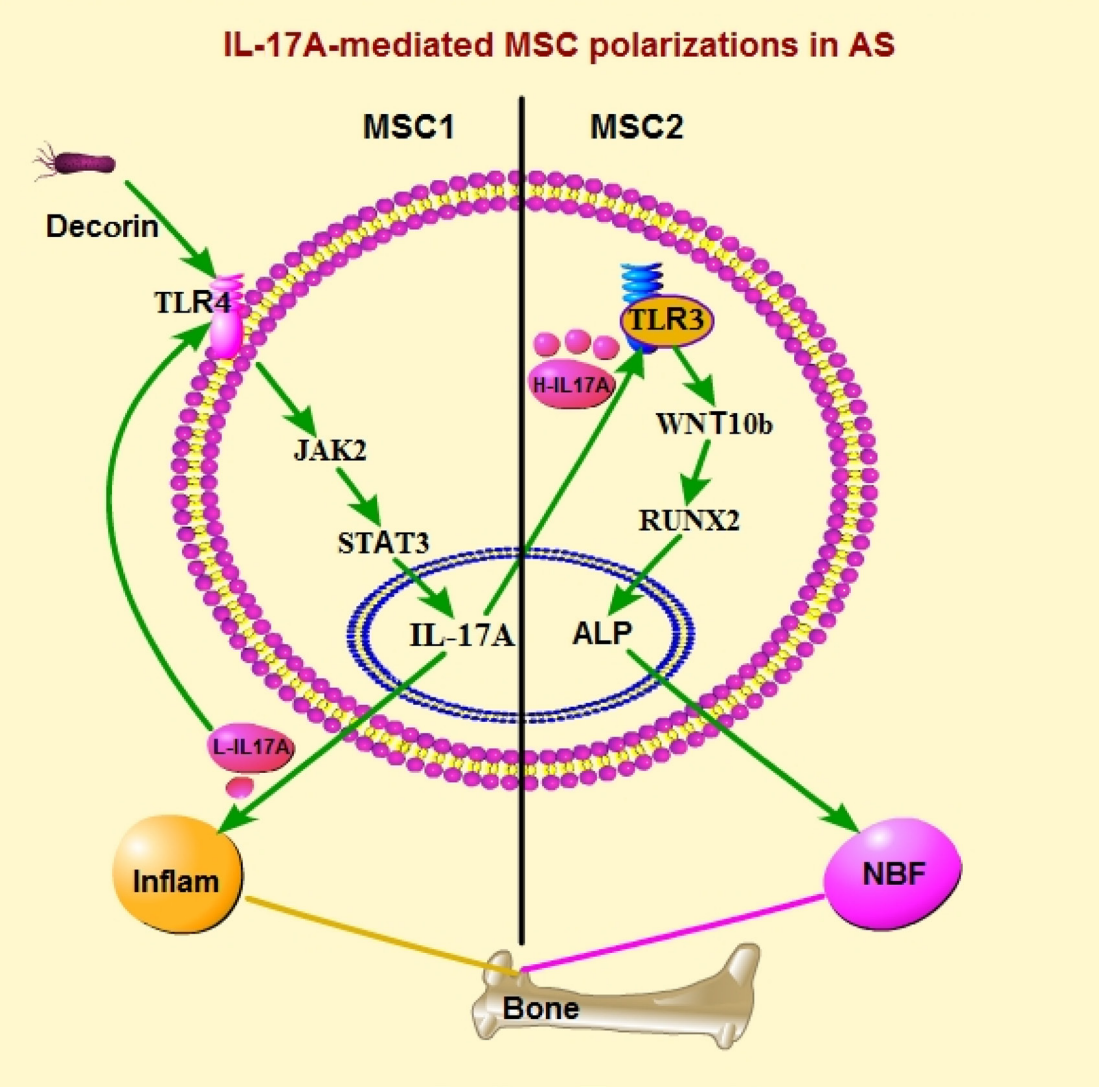
IL-17A-mediated MSC polarizations in AS Inflam: inflammation; NBF: new bone formation; H-IL17A: high level of IL-17A; L-IL17A: low level of IL-17A.

Furthermore, our results in flow cytometry showed MSCs from NBF-AS patients had high expression of TLR4 (98.86%) and CD40 (77.5%) with feature of MSCs including CD34+ (71.9%), CD45+ (90.1%), CD90.2+ (93.3%) and CD105^low^ (29.75%) (Figure 5H). However, MSCs from NBF+AS patients were TLR3^high^ (32%), TLR4– (0.9%) and CD40- (0.1%) with CD44+ (65.4%), CD90.2+ (98.5%) and CD105+ (98.4%) (Figure 5I and Supplementary Figure 5D). The level of supernatant IL-17A of MSCs was significantly higher in NBF-AS group than in both NBF+AS group and Healthy Donors group (both *p <* 0.01, Figure 5J). And the level of serum IL-17A was significantly higher in NBF-AS group than in both NBF+AS and Healthy Donors group (*p <* 0.05 and 0.01, respectively, Figure 5K). Furthermore, the relative mRNA expression of JAK2 and STAT3 was significantly higher, but that of PTGS1 and WNT10B was significantly lower in PBMCs of NBF-AS group than in that of Healthy Donors group (*p <* 0.01, 0.01, 0.05 and 0.01, Figure 5G). And IL17A, IL23R, TNF, IL10, CXCL10 and WNT10B had significantly higher relative mRNA expression in hip ligament of NBF+AS group than in that of Healthy Donors group (*p <* 0.01, 0.01, 0.05, 0.05, 0.05 and 0.05), but IL23R, TNF, CCL5, JAK2 and STAT3 had significantly higher relative mRNA expression in hip ligament of NBF-AS group than in that of Healthy Donors group (*p <* 0.05, 0.01, 0.05, 0.01 and 0.05), and STAT3 had significantly higher and WNT10B had significantly lower relative mRNA expression in hip ligament of NBF-AS group than in that of NBF+AS group (*p <* 0.01 and 0.05, Figure 1H). All these results showed MSC1 polarization promoted inflammation and peripherally high level of IL-17A of AS patients through JAK2/STAT3 pathway, while MSC2 polarization up-regulated by high level of IL-17A at local site promoted NBF of AS patients through WNT10B/RUNX2 pathway. The mechanism that low and high level of IL-17A promoted MSC1 and MSC2 polarization in AS respectively was showed in Figure 6 using software Pathway Builder 2.0 (Protein Lounge, USA).

## DISCUSSION

MSCs had both proinflammatory and anti-inflamatory, and both immune-enhancing and suppressing effects due to different secretory spectrum, and had distinctly different differentiation (adipocytic or non-adipocytic), but it’s unknown if different level of proinflammatory cytokine induced polarized differentiation of MSCs by activating different TLRs of MSCs [3, 4, 20]. By stimulating murine BMSCs *in vitro*, our study showed low level of IL-17A just like decorin promoted MSC1 polarization, which was TLR4^High^TLR3^low^ in flow cytometry with suppressed osteogenic and promoted adipocytic differentiation. However, we found high level of IL-17A just like dsRNA promoted MSC2 polarization, which was TLR4^low^TLR3^High^ with promoted osteogenic and suppressed adipocytic differentiation, and both MSC1 and MSC2 had good proliferating capability (Figure 1). It’s reported TLR4 and TLR3 activate JAK/STAT and WNT pathway in stem cells [16, 19] (that can induce Runx2-mediated TGFβRI expression in MSCs [17, 22]), resectively, and JAK/STAT and WNT pathways were antagonistic [8, 17, 18, 21]. Therefore, we want to know if it’s JAK/STAT and WNT pathways that determine the direction of MSC polarizations. Our results showed MSC1 primed by low level of IL-17A or decorin had high expression of JAK2, STAT3, IL17A and CCL5 with low expression of WNT10b, TGFb1, CXCL10 and ALP, while MSC2 primed by high level of IL17A had low expression of JAK2, STAT3, IL17A and CCL5 with high expression of WNT10b, TGFb1, CXCL10 and ALP. Furthermore, we found MSC1 had low expression of both JAK2 and STAT3, and secretion of IL-17A, TNF-α and CCL5 after inhibition of JAK2, and MSC2 had low expression of both WNT10b and RUNX2, and production of ALP, TGF-b1, CXCL10 and PGE_2_ after inhibition of WNT10b (Figure 3). Therefore, our results showed low level of IL-17A or decorin promoted MSC1 polarization through JAK2/STAT3 pathway, while high level of IL-17A promoted MSC2 polarization through WNT10b/RUNX2 pathway.

It’s reported that Th17 was the main source of IL-17A [8], but it’s still unknown if Th17/Treg polarizations were able to be mediated by MSC1/2 and M1/2 polarizations. By using coculture analysis *in vitro* to simulate the niche of MSCs *in vivo*, we found MSC1 promoted Th17 through promoting M1 and was promoted by Th17 and M1 in incontact way, while MSC2 promoted both Treg and M2, and was promoted by Treg and M2 in incontact way. Furthermore, we found MSC1 secreted IL-17A and CCL5 to promote M1 (that promoted IL-17A-producing Th17) through JAK2/STAT3 pathway, and MSC2 secreted TGF-β1 and expressed Cxcl10 and Ptges to promote M2 and Treg through WNT10b/RUNX2 pathway (Figure 4). These results not only further proved IL-17A promoted MSC1 polarization (Figure 1), but also surpported MSCs were able to be promoted by high level of TGF-β1 to keep self-renewal and regeneration [22].

Up to now, it’s difficult to find when NBF arise in chronic AS, which made the diagnosis of AS often delayed [29]. And because anti-inflammatory therapy was unable surely to retard development of NBF [30], it’s still unsure if NBF comes from inflammation in AS. It’s reported onset of AS was related to environmental factors such like gram-negative bacteria with LPS or mechanical stress [1], which lead to cartilage damage and release of PG that was able to stimulate pro-inflammatory TLR4 on many cells like MSCs [5, 31], and also used to make AS model [23, 24]. Decorin, a small molecule weight of PG, was able to induce both arthritis [32, 33] and fibrosis [34], so we used decorin that promoted MSC1 polarization (Figure 1 and Figure 2) to challenge murine and find if NBF of AS was a result of MSC2 polarization promoted by high level of IL-17A. Our results showed BALB/c female mice after receiving decorin at 3 months old only showed extensive inflammation before 6 months old, but after 6 months old NBF developed with extensive chronic inflammatory tissue hyperplasia including ankylosing joint, psoriasis-like skin disease, inflammation of eyes including blepharoconjunctivitis and iridocyclitis, and inflammatory cardiomyopathy. Furthermore, our results of flow cytometry and IHC showed MSC2 polarization promoted NBF and suppressed inflammation by promoting M2 and Treg polarizations, and the results of RT-qPCR showed decorin induced peripherally high expression of Il17a in early inflammation, while locally high level of IL-17A was related to NBF with up-regulated WNT10/RUNX2 pathway and down-regulated JAK2/STAT3 pathway in PGISp mice (Figure 4 and Supplementary Figure 4, and Supplementary Videos 1 and 3).

To find if NBF of AS patients was related with MSC2 polarization, we compared NBF-AS patients with NBF+AS patients and healthy donors, and found NBF of AS appeared at the site of inflammation that was related to hereditary IL-17A. Furthermore, we found NBF-AS patients showed MSC1 polarization with up-regulated JAK2/STAT3 pathway and high serum of IL-17A (without high expression at local site), while NBF+AS patients showed MSC2 polarization with up-regulated WNT10b/RUNX2 pathway and high expression of IL17A at local site (without high serum of- IL17A) (Figure 5 and Supplementary Figure 5).

## MATERIALS AND METHODS

### Patients

Eligible adult patients with a diagnosis of AS according to Assessment of Spondyloarthritis International Society classification criteria were chosen [1]. Exclusion criteria included a history of infection, metastatic cancer and usage of anti-inflammatory drug within 1 month. All of AS patients were HLA-B27 positive. NBF of AS was evaluated by both Bath Ankylosing Spondylitis Radiology Hip Index (BASRI-hip) [35] and radiographic ankylosing spondylitis spinal score (RASSS) [36]. The patients with BASRI-hip equal or more than 4 and RASSS more than 0 were Group NBF+AS and those with BASRI-hip equal or less than 3 and RASSS zero were Group NBF-AS. Severity of inflammation of AS patients was evaluated by both BASDAI and MRISIS. The overall progression of AS was decided by CTSIS. Group Healthy Donors included 5 patients with hip osteoarthritis (OA) scheduled to undergo THA and 5 patients with knee OA scheduled to undergo arthroscopy who were otherwise healthy. Background of three groups was comparable (Supplementary Table 1). All participants’ samples were collected from the orthopedic unit at Changhai Hospital at the time of diagnosis, but bone marrow (BM) cells were harvested by aspiration from the iliac crest from 5 patients of both Group NBF-AS and Group NBF+AS under general anesthesia, and some sample of ligament of hip joint got from 3 patients of Group NBF-AS, NBF+AS and Healthy Donors during THA. Fasting peripheral blood samples (5 ml) were collected from all AS patients and controls after the blood has coagulated. Within 4 hours, the serum aliquots were centrifuged at 2,500 rpm for 5 min and stored at –80^o^C for < 2 years with a single thaw before use. The levels of erythrocyte sedimentation rate (ESR) were measured by conventional Westergren method (Hitachi, Japan). The levels of C reactive protein (CRP) were measured using immuno-turbidimetry (Beckman Coulter, USA). The study was approved by the ethics committee of Second Military Medical University and written informed consent for both study participation and publication of identifying information/images (when applicable) was obtained from all individuals included in the study, and it was performed in accordance with the 1964 Declaration of Helsinki.

### Mice

Four weeks old BALB/c female mice (Shanghai Slac Laboratory Animal Co.) were maintained in clean facility because the germ-free condition prevented from appearances of AS mice [37]. All animal experiments were performed in compliance with the guide for the care and use of laboratory animals and were approved by the institutional biomedical research ethics committee of the Shanghai Institutes for Biological Sciences, Chinese Academy of Sciences. The mice were kept under natural light, with 6 mice each cage. Food and water were freely available, the room temperature was kept at 25 ± 3°C, and the ventilation was good. Mice were fed adaptively for one week and starved 1 day before the experiment. A total of 30 mice were used at the age of 8 weeks old for isolation of MSCs, PBMCs and Tc. Six mice at > 3 months old were used for PGISp model making and 6 mice were randomly selected as the control group.

The PGISp mouse model was made based on previous studies [23, 24]. A certain amount of decorin (Sigma, USA) was dissolved in 1x phosphate-buffered saline (PBS) at a concentration of 2 mg/ml. The mixture was stirred at 4°C and kept in a refrigerator at 4°C overnight. The mixture was emulsified with Freund’s Complete Adjuvant (FCA) (Sigma) or Freund’s Incomplete Adjuvant (FIA) (Sigma) in 1:1 (vol: vol) mixture in an ice water bath to prepare a decorin emulsion. Each mouse was injected intraperitoneal (i.p.) with 5 mg/kg decorin, together with 5 ml/kg FCA at 0, 6 week and 5 ml/kg FIA at 3 week. Control WT mice were injected i.p. with 10 ml/kg PBS. Each mouse was analyzed individually by weighting and taking video weekly. Three PGISp and control mice were collected for analysis of histology studies, gene expression, and fluorescence activated cell sorting (FACS) of bone marrow-derived MSCs (BMSCs), macrophage and Tc at 7 month.

All hind paws of mice were tested for mobility by gentle stretching weekly. Stiffness was assigned a score between 0 (normal) and 3 (completely immobile) [38]. Stiffness scores of 2 or higher were positive for ankylosing. The severity of the psoriasis-like skin condition on each week were assessed using 2 elements of PASI, to assign a score of 0–4 (0, none; 1, mild; 2, moderate; 3, severe; 4, very severe) for each of the parameters erythema and scaling [39]. Mice were also checked by subsequent MicroPET-CT scan. Three PGISp and control mice were euthanized from 7 months old and the psoriasis-like skin, inflammatory eye balls, heart and skeleton were immediately excised. A 4 mm punch biopsy of lesional skin, whole eye ball and heart were fixed in 10% neutral buffered formalin solution (Sigma) and paraffin embedded for histological analysis. Some lower lumbar spine and ankylosing joints were fixed in neutral buffered formalin. The remaining tissue was snap frozen in liquid nitrogen and stored at −80°C for subsequent analysis of RT-qPCR. Other females were observed regularly until 11 months old.

### Histology

Three PGISp or control WT mice were sacrificed at 7 month old, and the mouth skin, lumbar spine, foot joints, eyes and heart were dissected and immediately fixed with 10% neutral formalin for 12 h, dissolved in 2,000 ml PBS with 200 g 10% ethylenediaminetetraacetic acid (EDTA)-2Na (Gibco), and adjusted to pH 7.2-7.4. Decalcification treatment was conducted twice a week. Considerable work included decalcification for two months, gradient ethanol dehydration at different concentrations, sectioning of paraffin-embedded tissue (4 μm), and HE staining. Changes of the tissue (i.e. bone, cartilage, skin, eyes and heart) damage including inflammatory cell infiltration, hyperemia, hyperplasia, ossification, and other pathological changes were observed under light microscopy after adjustment of white balance and images were taken. For immunostaining, the sections were incubated for 1 hour at room temperature with monoclonal antibody (mAb) to mouse TLR3, TLR4, F4/80 (all 1: 100, Biolegend), or CD206 (1 : 100, Proteintech). Goat anti-mouse IgG2b conjugated with horseradish peroxidase (HRP, 1: 500, Dako). Following steps were performed using the immunostaining kit (Envision peroxidase detection system, DAKO) according to the manufacturer’s instructions. Images were observed using Axio Imager A2 (Zeiss, Germany). Then, the Image pro plus 6.0 (Media Cybernetics, USA) was used to analyze the IOD of immunohistochemical staining.

### Cell culture

Human and murine BMSCs were isolated from BM as previously described [14, 40]. BMSCs were cultured in complete culture medium (CCM) consisting of Dulbecco’s Modified Eagle’s Medium (DMEM, Gibco, USA), 10% (vol/vol) fetal bovine serum (FBS) (Gibco), 100 units/ml penicillin (Gibco) and 100 mg/ml streptomycin (Gibco) (1% PS). Cells were washed with PBS and the adherent viable cells were harvested using 0.25 % trypsin and 1 mM EDTA (Gibco) for 3–4 min at 37°C, reseeded at 100–200 cells/cm^2^ in CCM and incubated for 6–7 days (with medium change every 2–3 days) before freezing in DMEM containing 30% FBS and 5% dimethylsulfoxide (Sigma). BMSCs were used within four passages. MSC1/2 was induced by using improved methods [3]. Typically, MSCs were grown to 60–70% confluency in CCM prior to the start of an experiment. LPS-EB (10 ug/ml) (Invitrogen, USA) and poly (I: C) (30 ug/ml) (Invitrogen) were used as the control agonists for TLR4 and TLR3, respectively and added to fresh CCM and incubated with murine MSCs for 6 hours. To see effect of decorin and IL-17A on MSC1 and MSC2 polarizations, murine MSCs were seeded on 24-well multiplates and treated by decorin (8 ug/ml) [33] or 10 or 100 ng/ml recombinant murine IL-17A (Peprotech, USA) for 12 hours, respectively and then MSCs stimulated by 100 ng/ml IL-17A were treated by 20 ug/ml anti-IL-17A (Biolegend, USA) for 48 hours [13, 14]. To find the effect of inhibitors of pathway on MSC1 and MSC2, murine MSCs after stimulation of 10 ng/ml IL-17A were treated by inhibitor of JAK2/STAT3 pathway AG490 (Tyrphostin B42) (20 uM) (Selleckchem, USA) for 20 minutes. However, MSCs after stimulation of 100 ng/ml IL-17A were treated by inhibitor of Wnt/b-catenin pathway XAV-939 (10 uM) (Selleckchem, USA) for 30 minutes. After treatment MSCs were replaced by fresh CCM and used for subsequent analysis after 48 hours of culture. Following stimulation or treatment, supernatant from the first wash was further clarified by centrifugation at 10,000 × g for 10 min at 4°C, aliquoted and stored for further cytokine analysis at –80°C. The supernatant from the second wash was discarded and cell pellets were collected and assayed as described for the experiments.

Macrophages were differentiated from PBMCs and then polarized using an improved method [41]. PBMCs were obtained from venous blood and isolated using routine density centrifugation with Ficoll-Paque (Dakewe Biotech, China), and then cultured for 7 days in RPMI-1640-containing macrophage colony-stimulating factor (100 ng/ml) (Peprotech, USA) supplemented with 10% FBS and 1% PS. After differentiation, the cells (M0) were washed and cultured with polarization medium for 48 hours. M1 and M2 polarization medium contained 240 ng/ml LPS and 20 ng/ml of recombinant murine IL-4 (Peprotech) respectively in growth medium. Following polarization, the supernatant was collected and the cells were analyzed or used for co-culture. CD4+T cells were isolated from murine spleen by MACS using a CD4+T cell isolation kits (Miltenyi Biotec, USA) according to the manufacturers’ instructions. Murine CD4+T cells (1 × 10^6^ per well) were precultured on 24-well multiplates under RPMI-1640 medium (Gibco) supplemented with 10% FBS and 1% PS for 2–3 days. For Treg differentiation, recombinant mouse TGF-β1 (5 ng/ml) (R&D Systems, USA) were added. For Th17 differentiation, recombinant mouse IL-23 (30 ng/ml) (R&D Systems, USA) and TGF-β1 (2 ng/ml) were added [42, 43].

Cell viability analysis for live and dead cells was made by using Trypan blue stain, and performed by adding 10 ul of diluted cells with the concentration of 1–5 × 10^6^ cells/ml to 10 ul of a 0.4% Trypan blue solution (Life Technology, USA). Cell counts was obtained from automated cell counter (Countess, Invitrogen, USA), and the total number of viable cells (VC) was calculated by subtracting dead cell counts from total cell counts (TC). The percentage of viability of cells was calculated by the equation: (VC/TC) ×100%. Cell cycle analysis of mBMSCs was determined by measuring DNA content using PI dye (Sigma). Cells after culture were harvested and fixed with 70% ethanol and subjected to overnight incubation at –20°C. Fixed cells were washed with 1xPBS and incubated with 0.5 ml staining buffer which consisted of 10 mg/ml PI and 10 mg/ml RNase (Sigma) in 1xPBS for 30 min. At least 1 × 10^4^ cells were acquired and the percentages of phase G0/G1, S and G2/M were analyzed by FACSCalibur (BD Biosciences, USA). Cell proliferation test of MSCs was made by using cell counting kit-8 (CCK-8) (Biolite Biotech, China) and measuring the absorbance at 450 nm using a 96-well plate reader (Thermo Fischer Scientific, USA) according to the manufacturer’s protocol. MSC tri-lineage differentiation protocols were modified by plating 6 × 10^4^ P3 cells per well in 24-well tissue culture plate in triplicate. For osteogenic differentiation of MSCs, cells were cultured in osteogenic differentiation medium (Sigma) for 14 days and harvested for fixation, embedding in paraffin and staining with Alizarin Red S (Sigma). For adipocytic differentiation of MSCs, cells were cultured in adipogenic differentiation medium (Sigma) for 14 days and stained with Oil Red O (Sigma). For chondrocytic differentiation, MSCs were placed into chondrogenic medium (Sigma), and were stained with Allison blue (Sigma). ALP was analyzed by using Alkaline Phosphatase (AKP/ALP) Detection Kit and BCA Protein Assay Kit (Nanjing Jiancheng Bioengineering Institute, China) according to the manufacturers’ instructions. Cells were collected and lysed with 0.2% Triton X-100 (Sigma). Absorbance of ALP was measured spectrophotometrically at 520 nm on a micro plate reader. Standards, samples and BCA working reagents were added to micro plates in triplicates, and read using 562 nm absorbance. The activity of ALP was calculated as U/g protein (U/gprot). The phagocytic analysis for P2 macrophages was made by using CytoSelect Phagocytosis kit (Cell Biolabs, USA) and measuring the absorbance at 450 nm. Because LPS was able to promote differentiation of osteoclast (OC) from PBMCs [44], the differentiation of OC in M polarization was made by using TRAP kit (Genmed Scientifics Inc., USA) according to the manufacturer’s instructions. Stained cells were visualized under the microscope (Nikon Eclipse, Japan).

### Co-culturing of MSCs, macrophage and T cells

Transwell co-culturing analysis was based on previous study [41]. Murine M1/2 or Th17/Treg was seeded into a 12-well plate. The next day, the transwell inserts (upper chamber) (Translucent, High Density polycarbonate membrane, 0.4-mm-pore size, Corning) containing 1 × 10^5^ murine MSC1/2 were placed into the 12-well plate on upper chamber with the M1/2 (1:1 ratio) or Th17/Treg (1:50) that were initially seeded on lower chamber, respectively. On the other situation 1 × 10^5^ MSC1/2 were placed into the 12-well plate on upper chamber with M1/2 (1:1) that were initially seeded on upper chamber and the Th17/Treg (1:50) that were initially seeded on lower chamber, respectively. And M1/2 were placed into the 12-well plate on upper chamber with the Th17/Treg (1:50) that were initially seeded on lower chamber, respectively. The supernatants and cells were collected from each well after 48 hours of co-culture for subsequent analysis.

### Western blotting

Murine BMSCs were collected by lysing cells in radioimmunoprecipitation analysis buffer (50 mM Tris, pH 7.4, 150 mM NaCl, and 1 mM EDTA), 0.1% SDS, 1% TritonX-100, 1% sodium deoxycholate, and 1 mM phenylmethylsulfonyl fluoride (PMSF) (Amresco, USA). The protein levels were determined using a BCA assay kit (Pierce, USA). Each sample was size-fractionated using SDS-polyacrylamide gel electrophoresis (PAGE) and electrotransferred onto polyvinylidene difluoride (PVDF) transfer membranes (Millipore, USA). Blots were incubated for 1 h at room temperature in 5% skim milk for blocking, and proteins were detected with primary antibodies overnight at 4°C, and then blotted with horseradish peroxidase conjugated secondary antibodies (Supplementary Table 2) for 1 h at room temperature. The immunoblots were visualized using enhanced chemiluminescence (ECL) detection system (Millipore, USA). Equivalent loading was confirmed using an antibody against GAPDH. The levels of target protein bands were densitometrically determined using Quantity Ones 4.4 (Bio-Rad Laboratories, USA). The variation in the density of bands was expressed as fold changes compared to that of control in the blot after normalization to GAPDH.

### RNA extraction and RT-qPCR

Total RNA was extracted from the indicated cells or tissues with TRIzol reagent (Invitrogen) according to the manufacturer’s instructions. RNA samples were reverse-transcribed into cDNA with a Transcriptor First Strand cDNA Synthesis Kit(Roche). The cDNA samples were amplified by real-time fluorescent quantitative PCR (RT-qPCR) with a FastStart Universal SYBR Green Master (Rox) (Roche) on an ABI PRISM 7900 HT cycler (Applied Biosystems). The experiment was carried out by two-step PCR standard procedure (pre-denaturation: 95°C for 10 min, PCR reaction: 95°C for 15 s and 60°C for 60 s for 40 cycles). Sets of primers (Supplementary Table 2) were obtained from Sangon Biotech Co (China). Expression was normalized to human or mouse β-actin and presented as relative expression to control group by the 2^–ΔΔCt^ method.

### Flow cytometry

Flow cytometry was performed on freshly enzymatically treated samples using FACS Aria II (BD Biosciences). Cells were trypsinised, washed with FACS buffer (PBS plus 0.5% bovine serum albumin) and incubated with antibody for 30 min at 4°C. The proportion of MSC1 (TLR4+TLR3-CD40L+), MSC2 (TLR4-TLR3+CD40L-), M1 (F4/80+CD206-), M2 (F4/80+CD206+), Th17 (CD4+IL17+), Tregs (CD4+CD28+CD25+) was gated [3, 4] and calculated relative to total live cells. All antibodies (Supplementary Table 2) were used at the concentrations recommended by the manufacturers, with matched isotype controls and FlowJo software 10.0 were used for data acquiring and analysis.

### Enzyme-linked immunosorbent assay (ELISA)

For detection of cytokines in serum and supernatants of cell cultures, commercial ELISA kits for human and mouse IL-17A (Biolegend) with mouse IL-23 (Biolegend), TNF-α, CCL5, CCL10, PGE_2_ and TGF-β1 (all Senxiong Biotech, China) were used according to the manufacturers’ instructions. For all assays, optical density was determined on a plate reader (Thermo Fischer Scientific, USA) at an absorbency of 450 nm with wavelength correction at 540 nm for the optical imperfections on the plate.

### Observation of eyes in SLM

The PGISp and control WT mice at 5 month were anesthetized by an i.v. administration of 250 mg/kg chloral hydrate. Ten minutes after injection the eyes were examined by a single masked observer, who was an ophthalmologist, under slit lamp (SLM-3; Kanghua Science & Technology Co., Ltd., Chongqing, China). The inflammatory response was analyzed and based on the following parameters: ciliary hyperemia, central corneal edema, and peripheral corneal edema.

### Analysis of MicroPET-CT

The PGISp and control WT mice at 7 month were imaged using a Micro-PET-CT scanner (Pingseng Healthcare Co, China) according to the method of previous study [24]. The mice were anesthetized with 3% isoflurane in 100% oxygen at a flow rate of 1 L/minute (RWD life Science) and 7.4MBq 2-[18F] fluoro-2-deoxy-D-glucose (^18^F-FDG) was administered by lateral tail vein injection. One hour after injection, a static, ten-minute scan was acquired. Images were reconstructed using a maximum posteriori algorithm and three-dimensional spheroid regions of interest (ROIs) were drawn. The mean radioactivity in the ROIs were converted into percent of the injected dose per gram of tissue (%ID/g) by a calibrated cylinder factor and after division with the injected dose (corrected for residual and decay). Standardized uptake values (SUV) were obtained by multiplying the %ID/g of the ROI area by the weight of the animal.

### Statistical analysis

Data were expressed as mean ± SEM or count (percentage) unless otherwise specified. Each test was at least triplicate. Statistical significance between two groups was examined by the two-sided Student’s *t*-test or the Mann-Whitney test. Statistical work was performed using Prism 7.02 software (GraphPad, USA), and a *P* value of less than 0.05 was considered as significance.

## CONCLUSIONS

Our study showed NBF of AS was induced by MSC2 polarizations that was promoted by locally high level of IL-17A. In early phase of AS (without NBF), environmental factor (i.e. products of cartilage damage, decorin) activated TLR4+MSC1 to increase the level of IL-17A through JAK2/STAT3 pathway, and low level of IL-17A promoted TLR4+MSC1 polarization in a positive regulatory loop to induce inflammation. However, high level of IL-17A promoted TLR3+MSC2 polarization that promoted NBF in late phase of AS through WNT10b/RUNX2 pathway (Figure 6). So it’s important for control of NBF of AS to suppress not only locally high level of IL-17A (or keep reasonable level of IL-17A), but also over-MSC2-polarizaton at local site.

This study found (1) inflammation induced NBF in the new PGISp mouse model; (2) MSC1 polarization promoted by exogenous decorin and low level of IL-17A through JAK2/STAT3 pathway induced inflammation of PGISp; (3) MSC2 polarizations promoted by locally high level of IL-17A through WNT10b/RUNX2 pathway induced NBF of both PGISp mice and AS patients. This study brought the evidences that MSC2 polarization was related to multiple tissue hyperplasias such like NBF, which is important to develop polarized MSC therapy and modulators of MSC polarized differentiation like antibody of IL-17 or inhibitors of transduction signals for the treatment.

## SUPPLEMENTARY MATERIALS FIGURES, TABLES AND VIDEOS











## Abbreviations

AS: ankylosing spondylitis
NBF: new bone formation
PGISp: proteoglycan-induced spondylitis
WT: wild-type
MSC: mesenchymal stem cell
M: macrophage
PBMC: peripheral blood monocyte
Th17: T helper 17 cells
Treg: T regulatory cell
LPS: lipopolysaccharides
Poly(I:C): polyinosinic: polycytidylic acid
TLR: toll-like receptor
IL: interleukin
TNF: tumor necrosis factor
TGF-β: transforming growth factor beta
CCL: chemokines C-C motif ligand
CXCL: chemokines C-X-C motif
CCR: C-C motif chemokine receptor
PGE_2_: prostaglandin E2
IDO: indoleamine 2,3-dioxygenase
ALP: alkaline phosphatase
TRAP: tartrate-resistant acid phosphatase
WNT: wingless-type MMTV integration site family
RUNX: runt related transcription factor
JAK: Janus kinase
STAT: signal transducer and activator of transcription
MicroPET-CT: micro positron emission tomography computer tomography
PASI: psoriasis area severity index
IOD: integrated optical density
BASDAI: Bath ankylosing spondylitis disease activity index
MRISIS: magnetic resonance imaging sacroiliitis score
CTSIS: computer tomography sacroiliitis score
ESR: erythrocyte sedimentation rate
CRP: C reactive protein
RT-qPCR: real-time quantitative polymerase chain reaction

## References

[1] Dougados M, Baeten D (2011). Spondyloarthritis. Lancet.

[2] Agca R, Heslinga SC, van Halm VP, Nurmohamed MT (2016). Atherosclerotic cardiovascular disease in patients with chronic inflammatory joint disorders. Heart.

[3] Waterman RS, Tomchuck SL, Henkle SL, Betancourt AM (2010). A new mesenchymal stem cell (MSC) paradigm: polarization into a pro-inflammatory MSC1 or an Immunosuppressive MSC2 phenotype. PloS one.

[4] Bernardo ME, Fibbe WE (2013). Mesenchymal stromal cells: sensors and switchers of inflammation. Cell stem cell.

[5] Ashour DS (2015). Toll-like receptor signaling in parasitic infections. Expert review of clinical immunology.

[6] Ren G, Zhang L, Zhao X, Xu G, Zhang Y, Roberts AI, Zhao RC, Shi Y (2008). Mesenchymal stem cell-mediated immunosuppression occurs via concerted action of chemokines and nitric oxide. Cell stem cell.

[7] Li W, Ren G, Huang Y, Su J, Han Y, Li J, Chen X, Cao K, Chen Q, Shou P, Zhang L, Yuan ZR, Roberts AI (2012). Mesenchymal stem cells: a double-edged sword in regulating immune responses. Cell death and differentiation.

[8] Ebihara S, Date F, Dong Y, Ono M (2015). Interleukin-17 is a critical target for the treatment of ankylosing enthesitis and psoriasis-like dermatitis in mice. Autoimmunity.

[9] Galien R (2016). Janus kinases in inflammatory bowel disease: Four kinases for multiple purposes. Pharmacological reports.

[10] Cho DI, Kim MR, Jeong HY, Jeong HC, Jeong MH, Yoon SH, Kim YS, Ahn Y (2014). Mesenchymal stem cells reciprocally regulate the M1/M2 balance in mouse bone marrow-derived macrophages. Experimental & molecular medicine.

[11] Karin N, Wildbaum G (2015). The Role of Chemokines in Shaping the Balance Between CD4(+) T Cell Subsets and Its Therapeutic Implications in Autoimmune and Cancer Diseases. Frontiers in immunology.

[12] Nellimarla S, Mossman KL (2014). Extracellular dsRNA: its function and mechanism of cellular uptake. Journal of interferon & cytokine research.

[13] Han X, Yang Q, Lin L, Xu C, Zheng C, Chen X, Han Y, Li M, Cao W, Cao K, Chen Q, Xu G, Zhang Y (2014). Interleukin-17 enhances immunosuppression by mesenchymal stem cells. Cell death and differentiation.

[14] Osta B, Lavocat F, Eljaafari A, Miossec P (2014). Effects of Interleukin-17A on Osteogenic Differentiation of Isolated Human Mesenchymal Stem Cells. Frontiers in immunology.

[15] Mellett M, Atzei P, Bergin R, Horgan A, Floss T, Wurst W, Callanan JJ, Moynagh PN (2015). Orphan receptor IL-17RD regulates Toll-like receptor signalling via SEFIR/TIR interactions. Nature communications.

[16] Paracha RZ, Ahmad J, Ali A, Hussain R, Niazi U, Tareen SH, Aslam B (2014). Formal modelling of toll like receptor 4 and JAK/STAT signalling pathways: insight into the roles of SOCS-1, interferon-beta and proinflammatory cytokines in sepsis. PloS one.

[17] Tian A, Benchabane H, Wang Z, Ahmed Y (2016). Regulation of Stem Cell Proliferation and Cell Fate Specification by Wingless/Wnt Signaling Gradients Enriched at Adult Intestinal Compartment Boundaries. PLoS genetics.

[18] Phesse TJ, Buchert M, Stuart E, Flanagan DJ, Faux M, Afshar-Sterle S, Walker F, Zhang HH, Nowell CJ, Jorissen R, Tan CW, Hirokawa Y, Eissmann MF (2014). Partial inhibition of gp130-Jak-Stat3 signaling prevents Wnt-beta-catenin-mediated intestinal tumor growth and regeneration. Science signaling.

[19] Nelson AM, Reddy SK, Ratliff TS, Hossain MZ, Katseff AS, Zhu AS, Chang E, Resnik SR, Page C, Kim D, Whittam AJ, Miller LS, Garza LA (2015). dsRNA Released by Tissue Damage Activates TLR3 to Drive Skin Regeneration. Cell stem cell.

[20] Fakhry M, Hamade E, Badran B, Buchet R, Magne D (2013). Molecular mechanisms of mesenchymal stem cell differentiation towards osteoblasts. World journal of stem cells.

[21] Patterson AL, Pirochta J, Tufano SY, Teixeira JM (2017). Gain-of-function beta-catenin in the uterine mesenchyme leads to impaired implantation and decidualization. The Journal of endocrinology.

[22] Luo K (2017). Signaling Cross Talk between TGF-beta/Smad and Other Signaling Pathways. Cold Spring Harbor perspectives in biology.

[23] Haynes KR, Pettit AR, Duan R, Tseng HW, Glant TT, Brown MA, Thomas GP (2012). Excessive bone formation in a mouse model of ankylosing spondylitis is associated with decreases in Wnt pathway inhibitors. Arthritis research & therapy.

[24] Lin S, Qiu M, Chen J (2015). IL-4 Modulates Macrophage Polarization in Ankylosing Spondylitis. Cellular physiology and biochemistry.

[25] Garrett S, Jenkinson T, Kennedy LG, Whitelock H, Gaisford P, Calin A (1994). A new approach to defining disease status in ankylosing spondylitis: the Bath Ankylosing Spondylitis Disease Activity Index. The Journal of rheumatology.

[26] Hermann KG, Baraliakos X, van der Heijde DM, Jurik AG, Landewe R, Marzo-Ortega H, Ostergaard M, Rudwaleit M, Sieper J, Braun J, Assessment in SpondyloArthritis International Society (ASAS) (2012). Descriptions of spinal MRI lesions and definition of a positive MRI of the spine in axial spondyloarthritis: a consensual approach by the ASAS/OMERACT MRI study group. Annals of the rheumatic diseases.

[27] Gao D, Li KP, Wen QF, Zhu J, Zhang JL, Huang F (2016). [A preliminary exploration of low-dose semicoronal CT of the sacroiliac joints in the diagnosis of ankylosing spondylitis]. [Article in Chinese]. Zhonghua nei ke za zhi.

[28] Cortes A, Hadler J, Pointon JP, Robinson PC, Karaderi T, eo P, Cremin K, Pryce K, Harris J, Lee S, Joo KB, Shim SC, International Genetics of Ankylosing Spondylitis Consortium (IGAS) (2013). Identification of multiple risk variants for ankylosing spondylitis through high-density genotyping of immune-related loci. Nature genetics.

[29] Sorensen J, Hetland ML, all departments of rheumatology in Denmark (2015). Diagnostic delay in patients with rheumatoid arthritis, psoriatic arthritis and ankylosing spondylitis: results from the Danish nationwide DANBIO registry. Annals of the rheumatic diseases.

[30] Sieper J, Poddubnyy D (2016). New evidence on the management of spondyloarthritis. Nature reviews Rheumatology.

[31] Pollanen R, Sillat T, Pajarinen J, Levon J, Kaivosoja E, Konttinen YT (2009). Microbial antigens mediate HLA-B27 diseases via TLRs. Journal of autoimmunity.

[32] Salo J, Jaatinen A, Soderstrom M, Viljanen MK, Hytonen J (2015). Decorin binding proteins of Borrelia burgdorferi promote arthritis development and joint specific post-treatment DNA persistence in mice. PloS one.

[33] Merline R, Moreth K, Beckmann J, Nastase MV, Zeng-Brouwers J, Tralhao JG, Lemarchand P, Pfeilschifter J, Schaefer RM, Iozzo RV, Schaefer L (2011). Signaling by the matrix proteoglycan decorin controls inflammation and cancer through PDCD4 and MicroRNA-21. Science signaling.

[34] Kamma-Lorger CS, Pinali C, Martinez JC, Harris J, Young RD, Bredrup C, Crosas E, Malfois M, Rodahl E, Meek KM, Knupp C (2016). Role of Decorin Core Protein in Collagen Organisation in Congenital Stromal Corneal Dystrophy (CSCD). PloS one.

[35] Sieper J, van der Heijde D, Landewe R, Brandt J, Burgos-Vagas R, Collantes-Estevez E, Dijkmans B, Dougados M, Khan MA, Leirisalo-Repo M, van der Linden S, Maksymowych WP, Mielants H (2009). New criteria for inflammatory back pain in patients with chronic back pain: a real patient exercise by experts from the Assessment of SpondyloArthritis international Society (ASAS). Annals of the rheumatic diseases.

[36] Baraliakos X, Listing J, Rudwaleit M, Sieper J, Braun J (2009). Development of a radiographic scoring tool for ankylosing spondylitis only based on bone formation: addition of the thoracic spine improves sensitivity to change. Arthritis and rheumatism.

[37] Taurog JD, Richardson JA, Croft JT, Simmons WA, Zhou M, Fernandez-Sueiro JL, Balish E, Hammer RE (1994). The germfree state prevents development of gut and joint inflammatory disease in HLA-B27 transgenic rats. The Journal of experimental medicine.

[38] Sinkorova Z, Capkova J, Niederlova J, Stepankova R, Sinkora J (2008). Commensal intestinal bacterial strains trigger ankylosing enthesopathy of the ankle in inbred B10. BR (H-2(k)) male mice. Human immunology.

[39] Kjaer TN, Thorsen K, Jessen N, Stenderup K, Pedersen SB (2015). Resveratrol ameliorates imiquimod-induced psoriasis-like skin inflammation in mice. PloS one.

[40] He T, Chi G, Tian B, Tang T, Dai K (2015). Lentivirus transduced interleukin-1 receptor antagonist gene expression in murine bone marrow-derived mesenchymal stem cells *in vitro*. Molecular medicine reports.

[41] Freytes DO, Kang JW, Marcos-Campos I, Vunjak-Novakovic G (2013). Macrophages modulate the viability and growth of human mesenchymal stem cells. Journal of cellular biochemistry.

[42] Song X, Dai D, He X, Zhu S, Yao Y, Gao H, Wang J, Qu F, Qiu J, Wang H, Li X, Shen N, Qian Y (2015). Growth Factor FGF2 Cooperates with Interleukin-17 to Repair Intestinal Epithelial Damage. Immunity.

[43] Yang R, Liu Y, Kelk P, Qu C, Akiyama K, Chen C, Atsuta I, Chen W, Zhou Y, Shi S (2013). A subset of IL-17(+) mesenchymal stem cells possesses anti-Candida albicans effect. Cell research.

[44] Zheng M, Ge Y, Li H, Yan M, Zhou J, Zhang Y (2014). Bergapten prevents lipopolysaccharide mediated osteoclast formation, bone resorption and osteoclast survival. International orthopaedics.

