# Supplementary material for: Interleukin-17A-promoted MSC2 polarization related with new bone formation of ankylosing spondylitis
Source: Oncotarget. 2017 Sep 11;8(57):96993–7008. doi: 10.18632/oncotarget.20823 (PMC5722540; doi:10.18632/oncotarget.20823)
Supplement: Supplementary file 1 [file oncotarget-08-96993-s001.pdf]

Interleukin-17A-promoted MSC2 polarization related with new bone formation of ankylosing spondylitis

SUPPLEMENTARY MATERIALS

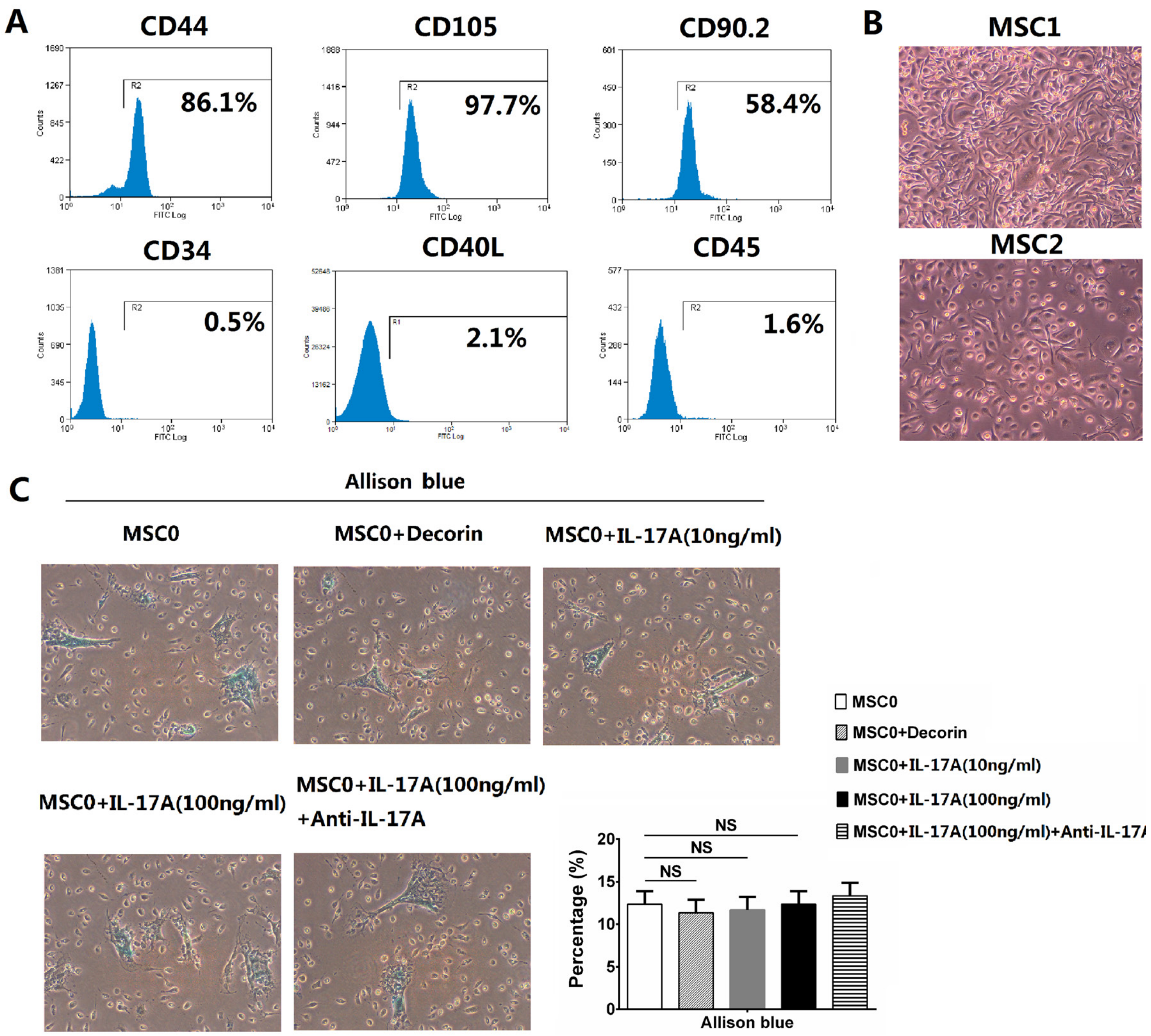

Supplementary Figure 1: Characteristics of MSC1 and MSC2 polarizations primed by low and high level of IL-17A, respectively. Values are the mean ± SEM (all *n* = 3), NS= no significance. (**A**) Flow cytometry of MSC0. (**B**) Microscopic view of MSC1 and MSC2 (orignal magnification 100×). (**C**) Chondrocytic differentiation of murine MSCs.

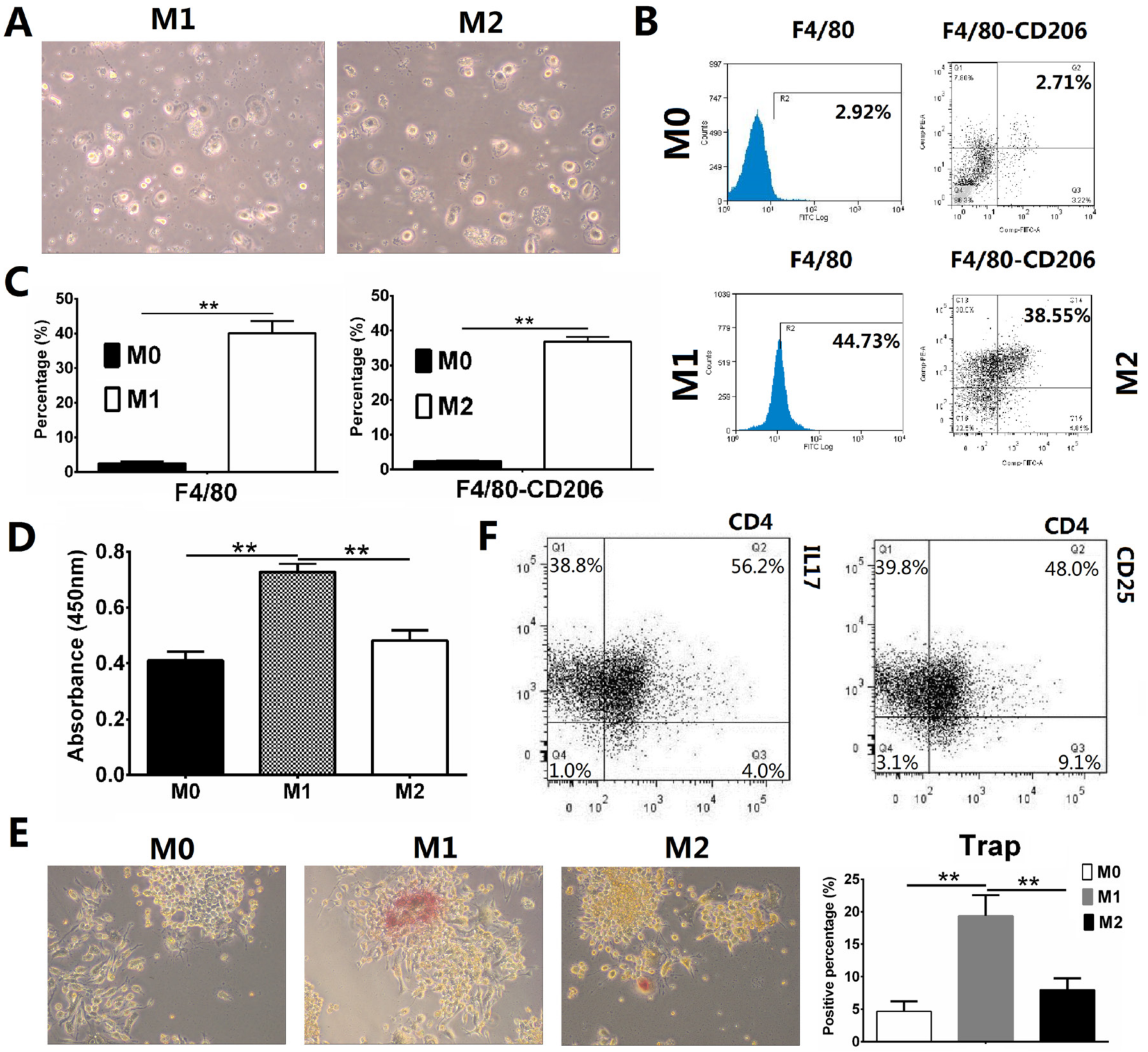

Supplementary Figure 2: Characteristics of polarized M1, M2, Th17 and Treg. Values are the mean ± SEM (all $n = 3$). $^{*}P < 0.05$, $^{**}P < 0.01$. (**A**) Microscopic view of LPS-chalenged murine macrophage (M1) and IL-4-chanlenged murine macrophage (M2) (Original magnification 100×). (**B**–**C**) Flow cytometry of M1 and M2. (**D**) Cellular phagocytosis assay for M0, M1 and M2. (**E**) TRAP staining analysis for M0, M1 and M2. (**F**) Flow cytometry of Th17 and Treg.

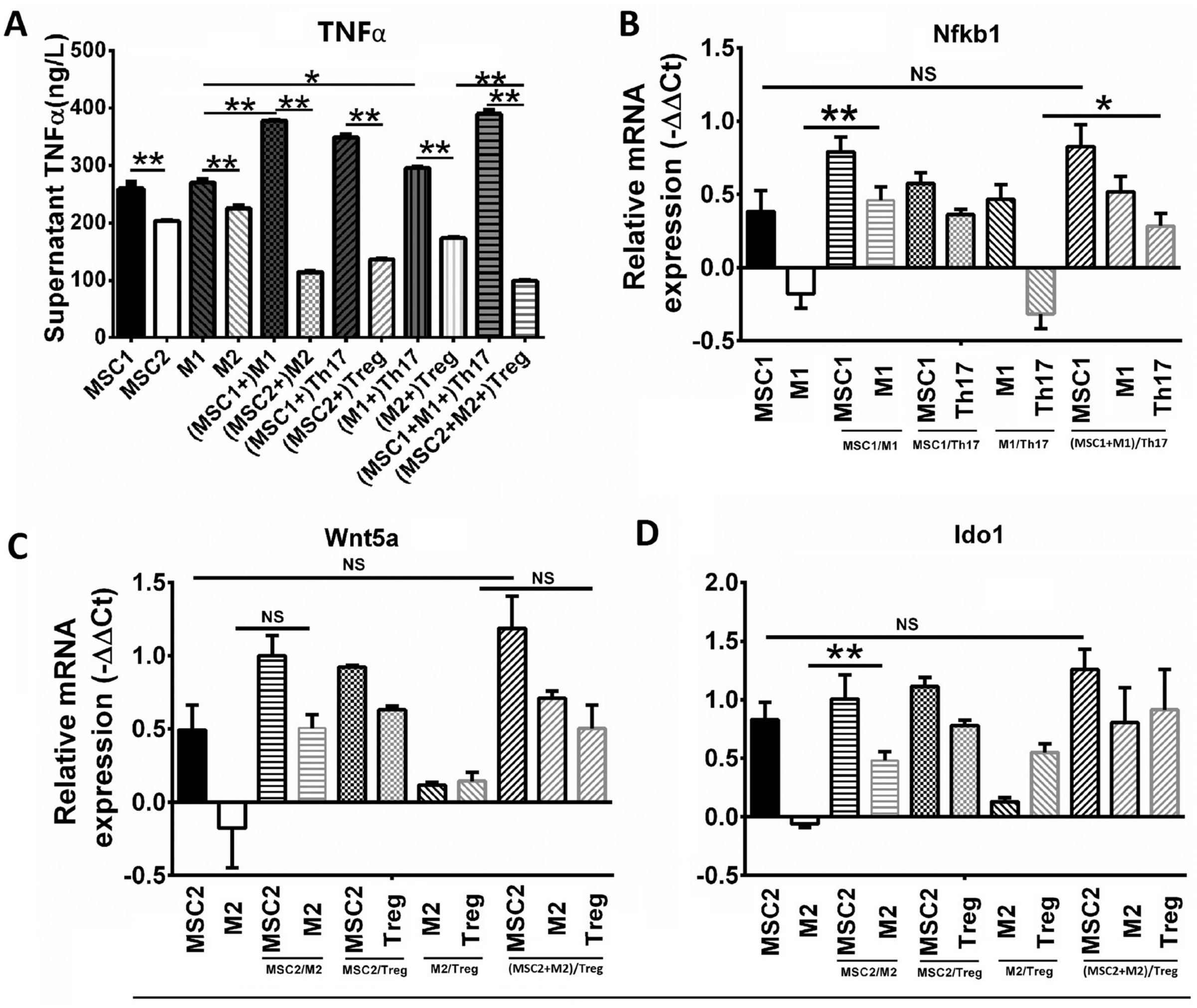

Supplementary Figure 3: Traditional signals related with MSC1/2 polarizations promoting M1/2 and Th17/Treg polarizations, respectively. Values are the mean ± SEM (all $n = 3$), NS = no significance. $^{*}P < 0.05$, $^{**}P < 0.01$. (**A**) The analysis for supernatant TNFα in Elisa. (**B**–**D**) The relative mRNA expression of Nfkb1 (B), Wnt5a (C) and Ido1 (D) in RT-qPCR.

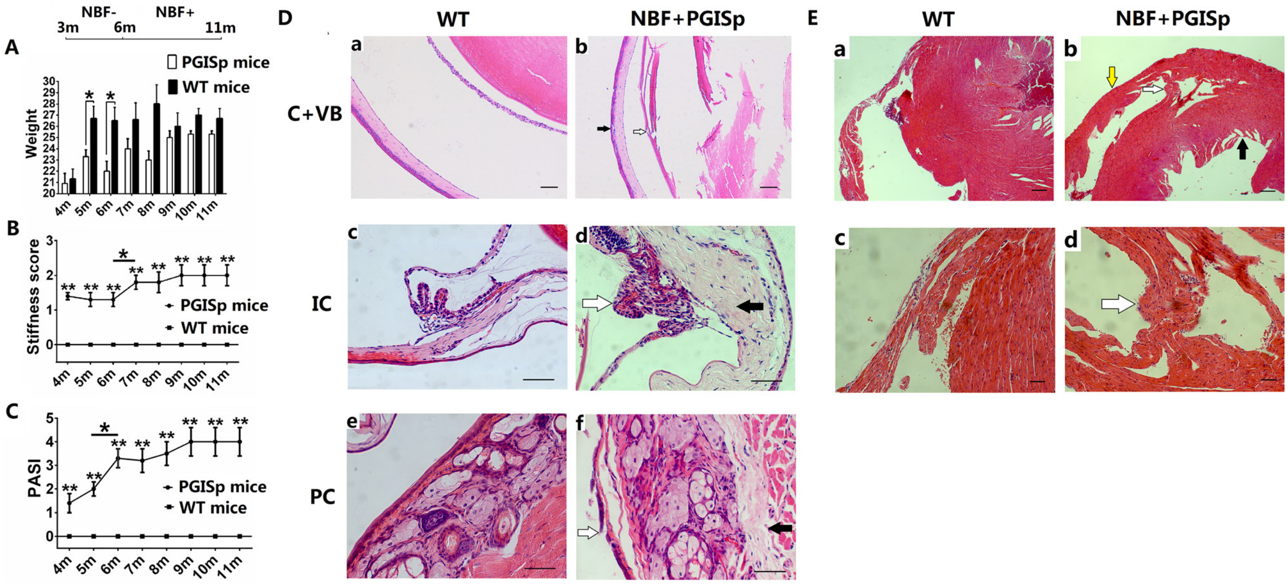

Supplementary Figure 4: PGISp mice with new bone formation showed extensive tissue hyperplasia in chronic inflammatory damage. Values are the mean ± SEM (all $n = 3$). $^{*}P < 0.05$, $^{**}P < 0.01$. (**A**–**C**) NBF+PGISp mice showed different feature in weight (A), Stiffness score (B) and PASI (C) compared to NBF-PGISp mice. (**D**–**E**) Tissue hyperplasia of NBF+PGISp mice in eyes (D) and heart (E) by HE staining.

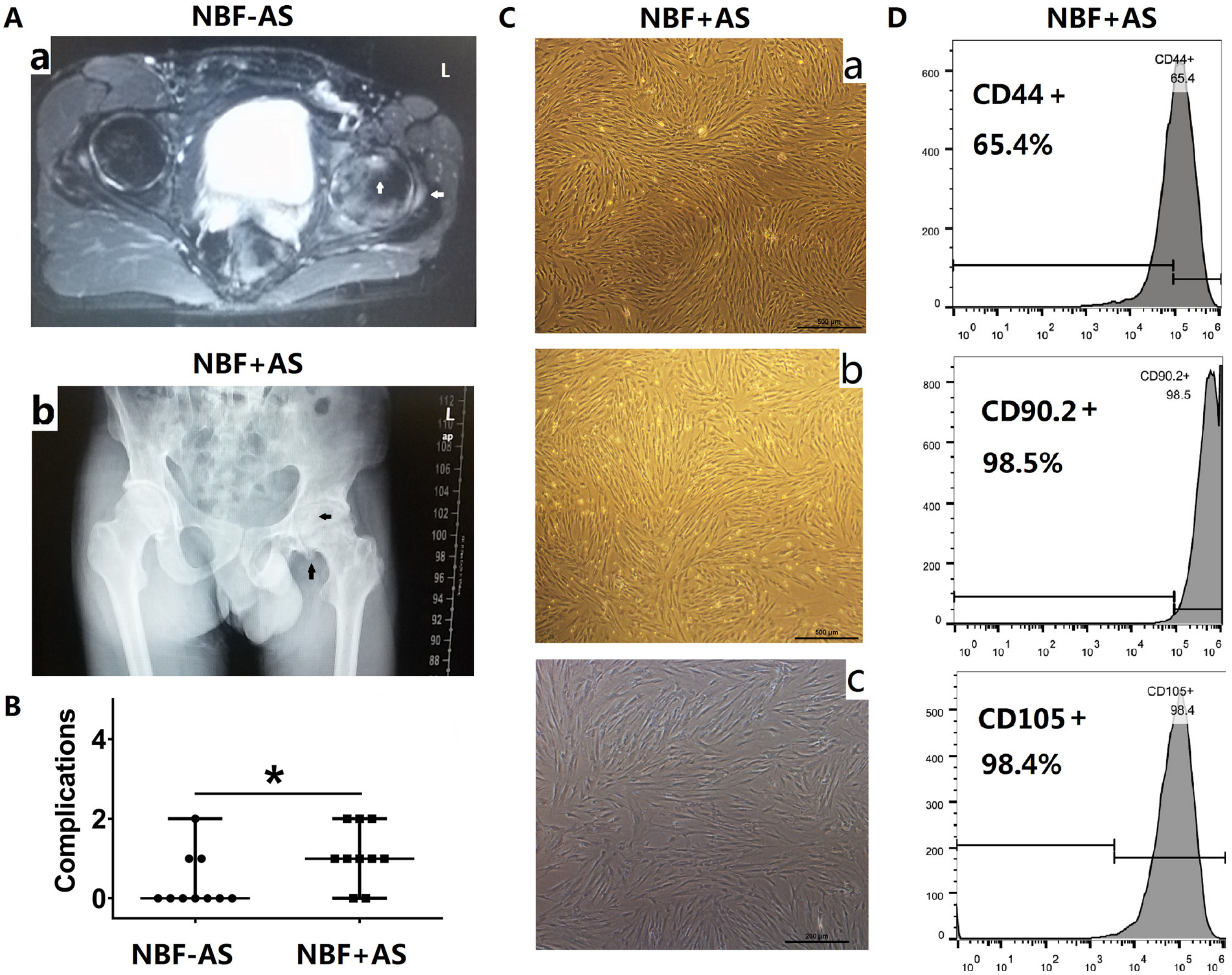

Supplementary Figure 5: Characteristics of AS patients with new bone formation. Values are the mean ± SEM (both $n = 10$). $^{*}P < 0.05$. (**A**) The radiological image of pelvis of an AS patient. (**B**) Complications of NBF- and NBF+AS patients. (**C**–**D**) Microscopic view (C) and flow cytometry (D) of MSCs from a NBF+AS patient.

Supplementary Table 1: Baseline characteristics of AS patients and healthy donors

Items	Group NBF-AS (N = 10)	Group NBF+AS (N = 10)	Group Healthy Donors (N = 10)
Age, mean (± sem) years	36.4 (± 2.5)	43.8 (± 3.7)	39.5 (± 4.6)
Sex, male (%)	8 (80%)	9 (90%)	6 (60%)
Marriage (%)	100%	80%	80%
Degree of education, median (range)	2 (1–4)	3 (1–4)	3 (1–4)
Place of birth, Southern (%)	80%	80%	80%
Precipitating factors (%)	20%	20%	
Family history (%)	30%	20%	
Age of initial pain, mean (± sem) years	18.3 (±1.8)	26.4 (± 4.5)	
Age of initial ankylosis, mean (± sem) years	28.0 (1.3)	35.4 (3.9)	
Duration of disease, mean (± sem) years	18.1 (3.2)	17.4 (3.2)	
Accompanied diseases*, median (range)	1(1–4)	1 (1–4)	
ESR (mm/H)	16.7 (± 2.3)	19.5 (± 3.1)	
CRP (mg/L)	19.6 (± 7.2)	12.6 (± 2.1)	

*Including hip or knee arthritis, dactylitis, enthesopathy, psoriasis, uveitis, inflammatory bowel disease or cardiovascular diseases.

Supplementary Table 2: List of primers and antibodies. See Supplementary_Table_2

Supplementary Video 1: A NBF-PGISp mouse. See Supplementary_Video_1
Supplementary Video 2: A NBF+PGISp mouse. See Supplementary_Video_2
Supplementary Video 3: NBF+PGISp and wild-type mice in long term observation. See Supplementary_Video_3
