# Supplementary material for: Interleukin-17A-promoted MSC2 polarization related with new bone formation of ankylosing spondylitis
Source: Oncotarget. 2017 Sep 11;8(57):96993–7008. doi: 10.18632/oncotarget.20823 (PMC5722540; doi:10.18632/oncotarget.20823)
Supplement: Supplementary file 2 [file oncotarget-08-96993-s002.doc]

**Supplementary Table 2:** List of primers and antibodies

|  | **Human primers** |
| --- | --- |
| **IL17A (CTLA-8)** | FORWARD-TACCTCAACCGTTCCACGTC  REVERSE-TTTCCCTCCGCATTGACACA |
| **IL23R (Interleukin 23 receptor )** | FORWARD-AAACGCACTAGGCATGGAAG  REVERSE-GGTCTTGGGCACTGTAGCAT |
| **TNF (Tumor necrosis factor )** | FORWARD-CTCCTCACCCACACCATCA  REVERSE-GGAAGACCCCTCCCAGATAG |
| **IL10 (Interleukin 10)** | FORWARD-CGAGATGCCTTCAGCAGAGT  REVERSE-CGCCTTGATGTCTGGGTCTT |
| **TGFB1 (Transforming growth factor beta 1)** | FORWARD-CGACTCGCCAGAGTGGTTAT  REVERSE-AGTGAACCCGTTGATGTCCA |
| **TGFB3 (Transforming growth factor beta 3)** | FORWARD-CGTGAGTGGCTGTTGAGAAG  REVERSE-CACCTCGTGAATGTTTTCCA |
| **CCL5 (C-C motif chemokine ligand 5 or RANTES)** | FORWARD-TGCTGCTTTGCCTACATTGC  REVERSE-CTTGTTCAGCCGGGAGTCAT |
| **CXCL10 (C-X-C motif chemokine ligand 10 or IP-10 )** | FORWARD- CCATTCTGATTTGCTGCCTTA  REVERSE- CTCACCCTTCTTTTTCATTGTAGC |
| **PTGS1 (Prostaglandin -endoperoxide synthase 1)** | FORWARD-CCAAAGGGAAGAAGCAGTTG  REVERSE-CTGGTGGGTGAAGTGTTGTG |
| **IDO1 (Indoleamine 2,3-dioxygenase 1)** | FORWARD-AGACTGCTGGTGGAGGACAT  REVERSE-CACGGACTGAGGGATTTGAC |
| **NFKB1 (Nuclear factor kappa B subunit 1)** | FORWARD-AAATGGGCTACACCGAAGCA  REVERSE-TTGCGGAAGGATGTCTCCAC |
| **JAK2 (Janus kinase 2)** | FORWARD-GTCCACCAGCGGAATTTATG  REVERSE-ATCTCATCTGGGCATCCATC |
| **STAT2 (Signal transducer and activator of transcription 2)** | FORWARD-GTCAGAAGGGGGCATTACCT  REVERSE-GATTTCAGTCAGCGGGAGTG |
| **STAT3 (Signal transducer and activator of transcription 3)** | FORWARD-TGGAGGAGAGAATCGTGGAG  REVERSE-TTTGACCAGCAACCTGACTTT |
| **WNT5A (Wingless-type MMTV integration site family, member 5A)** | FORWARD-CGGTGTACAACCTGGCTGAT  REVERSE-GCGCTGTCGTACTTCTCCTT |
| **WNT10B (Wingless-type MMTV integration site family, member 10B)** | FORWARD-GCAGTCGGGCTCTAAGCAAT  REVERSE-GATGTGCAGACCCTGAAGCG |
| **β-ACTIN** | CAT.No B661102 |
|  | **Mouse primers** |
| **Il17a (CTLA-8)** | FORWARD-CTCAGGCTTCCTTTGGAGATT;  REVERSE-TCCTTTCTGGGTTGTGTGGT; |
| **Il23a (Interleukin 23, alpha subunit p19)** | FORWARD-CCCGTATCCAGTGTGAAGATG;  REVERSE-AGATGTCAGAGTCAAGCAGGTG; |
| **Tnf (Tumor necrosis factor )** | FORWARD-CACCACCATCAAGGACTCAA;  REVERSE-GAGACAGAGGCAACCTGACC; |
| **Il6 (Interleukin 6)** | FORWARD- CGGAGAGGAGACTTCACAGAG;  REVERSE- ATTTCCACGATTTCCCAGAG; |
| **Il10 (Interleukin 10)** | FORWARD-CTTTGCTATGGTGTCCTTTCA;  REVERSE-ATCTCCCTGGTTTCTCTTCC; |
| **Tgfb1 (Transforming growth factor beta 1)** | FORWARD-ATTCCTGGCGTTACCTTGG;  REVERSE-AGCCCTGTATTCCGTCTCCT; |
| **Tgfb3 (Transforming growth factor beta 1)** | FORWARD-GAAGAGGGTGGAAGCCATTA;  REVERSE-GCTGTTGTAAAGTGCCAGGA; |
| **Ccl5 (C-C motif chemokine ligand 5 or RANTES)** | FORWARD-GTGCCAACCCAGAGAAGAAG;  REVERSE-AGCAAGCAATGACAGGGAAG; |
| **Cxcl10 (C-X-C motif chemokine ligand 10 or IP-10 )** | FORWARD-GCTCAGGCTCGTCAGTTCTAA;  REVERSE-CCTTGGGAAGATGGTGGTT; |
| **Ccr5 (C-C motif chemokine receptor 5)** | FORWARD-ACCCATTGAGGAAACAGCAA;  REVERSE-AGGTCTGAAGGCAGGAACAA; |
| **Ptges (Prostaglandin E synthase, or mPGES-1)** | FORWARD-CCTTGGGTCTTTGCCATACTT;  REVERSE-GCACTGGACTGGGTAGAACAG; |
| **Ido1 (Indoleamine 2,3-dioxygenase 1)** | FORWARD-CCAGTCCGTGAGTTTGTCATT;  REVERSE-ATCAGTGGGCTTCTTCTTCG; |
| **Nfkb1 (Nuclear factor kappa B subunit 1)** | FORWARD-TGGACGACTCTTGGGAGAAG  REVERSE-CACAGGCTCATACGGTTTCC |
| **Jak2 (Janus kinase 2)** | FORWARD-CAACCTCAGCGGGACTAAGA;  REVERSE-GGCAGCATTTGGTAAACTGG; |
| **Stat3 (Signal transducer and activator of transcription 3)** | FORWARD-GGAACAAGGTGAGGGCTTCT;  REVERSE-GCCAAGGAGAGGGAAAGTG; |
| **Wnt5a (Wingless-type MMTV integration site family, member 5A)** | FORWARD-ACGAATACCAGGAAGCAAGC;  REVERSE-CCAAAGAGAGGGTGGAGAAC; |
| **Wnt10b (Wingless-type MMTV integration site family, member 10B)** | FORWARD-TCCACTACAGCCCAGAACCT;  REVERSE-TCCCAAGAGCCTGACAAGAC; |
| **Runx2 (Runt related transcription factor 2)** | FORWARD-CGTCACCTCCATCCTCTTTC;  REVERSE-GCATCACAACAGCCACAAGT; |
| **β-ACTIN** | CAT.No B661302 |
| **Antibodies** |  |
| **Anti-mouse CD25-PE (Ly43)** | Biolegend, USA |
| **Anti-mouse CD34-APC (APG)** | Biolegend, USA |
| **Anti-mouse CD90.2-Alexa Fluor 488 (Thy1.2)** | Biolegend, USA |
| **Anti-mouse CD105-PE/Cy7 (Endoglin)** | Biolegend, USA |
| **Anti-mouse CD154-PerCP/Cy (CD40L)** | Biolegend, USA |
| **Anti-mouse CD284-APC (TLR4)** | Biolegend, USA |
| **Anti-mouse F4/80-FITC (EMR1)** | Biolegend, USA |
| **Anti-mouse CD206-PE (MMR)** | Biolegend, USA |
| **Anti-mouse IL-17A-Brilliant Violet 421 (CTLA-8)** | Biolegend, USA |
| **Anti-human CD40-PE (BP50)** | Biolegend, USA |
| **Anti-human CD90-PerCP/Cy5.5 (Thy1)** | Biolegend, USA |
| **Anti-human CD283-PE (TLR3)** | Biolegend, USA |
| **Anti-** **human CD284-APC (TLR4)** | Biolegend, USA |
| **Anti-human/mouse CD44-Alexa Fluro 488** | Biolegend, USA |
| **Anti-mouse CD4-FITC (L3T4)** | eBioscience, USA |
| **Anti-mouse CD45-FITC (LCA)** | eBioscience, USA |
| **Anti-human CD105-PE (Endoglin)** | eBioscience, USA |
| **Rabbit polyclonal anti-JAK2 [EPR108(2)]** | Abcam, UK |
| **Rabbit polyclonal anti-STAT3 [EPR787Y]** | Abcam, UK |
| **Rabbit polyclonal anti-WNT10b** | Abcam, UK |
| **Rabbit polyclonal anti-RUNX2** | Abcam, UK |
| **Mouse monoclonal anti-TGF beta 1 [2Ar2]** | Abcam, UK |
| **monoclonal mouse anti-GAPDH** | Kangchen, China |
